# Population Pharmacokinetics of Casirivimab and Imdevimab in Pediatric and Adult Non-Infected Individuals, Pediatric and Adult Ambulatory or Hospitalized Patients or Household Contacts of Patients Infected with SARS-COV-2

**DOI:** 10.1007/s11095-024-03764-5

**Published:** 2024-09-18

**Authors:** Kuan-Ju Lin, Kenneth C. Turner, Maria Rosario, Lutz O. Harnisch, John D. Davis, A. Thomas DiCioccio

**Affiliations:** Regeneron Pharmaceuticals, Inc, 777 Old Saw Mill River Road, Tarrytown, NY 10591 USA

**Keywords:** COVID-19, Monoclonal antibody, Pharmacodynamics, Pharmacokinetics

## Abstract

**Introduction:**

Casirivimab (CAS) and imdevimab (IMD) are two fully human monoclonal antibodies that bind different epitopes on the receptor binding domain of severe acute respiratory syndrome coronavirus 2 (SARS-CoV-2) and block host receptor interactions. CAS + IMD and was developed for the treatment and prevention of SARS-CoV-2 infections.

**Methods:**

A population pharmacokinetic (PopPK) analysis was conducted using pooled data from 7598 individuals from seven clinical studies to simultaneously fit concentration–time data of CAS and IMD and investigate selected covariates as sources of variability in PK parameters. The dataset comprised CAS + IMD-treated pediatric and adult non-infected individuals, ambulatory or hospitalized patients infected with SARS-CoV-2, or household contacts of patients infected with SARS-CoV-2.

**Results:**

CAS and IMD concentration–time data were both appropriately described simultaneously by a two-compartment model with first-order absorption following subcutaneous dose administration and first-order elimination. Clearance estimates of CAS and IMD were 0.193 and 0.236 L/day, respectively. Central volume of distribution estimates were 3.92 and 3.82 L, respectively. Among the covariates identified as significant, body weight and serum albumin had the largest impact (20–34%, and ~ 7–31% change in exposures at extremes, respectively), while all other covariates resulted in small differences in exposures. Application of the PopPK model included simulations to support dose recommendations in pediatrics based on comparable exposures of CAS and IMD between different weight groups in pediatrics and adults following weight-based dosing regimens.

**Conclusions:**

This analysis provided important insights to characterize CAS and IMD PK simultaneously in a diverse patient population and informed pediatric dose selection.

**Supplementary Information:**

The online version contains supplementary material available at 10.1007/s11095-024-03764-5.

## Introduction

Coronaviruses are a family of enveloped, single-stranded ribonucleic acid (RNA) viruses. In recent decades, two highly pathogenic strains of coronavirus were identified in humans: the severe acute respiratory syndrome coronavirus (SARS-CoV) and the Middle East respiratory syndrome coronavirus. These viruses were found to cause severe, and sometimes fatal, respiratory illness [1, 2]. In December 2019, pneumonia of an unknown cause was identified in clusters of individuals in Wuhan City, China [3]. A novel enveloped RNA betacoronavirus – severe acute respiratory syndrome coronavirus 2 (SARS-CoV-2) – was identified in these individuals, and the disease caused by SARS-CoV-2 infection was later designated as COVID-19 by the World Health Organization [4, 5].

Coronaviruses consist of an RNA genome packaged in nucleocapsid protein surrounded by an outer envelope. The envelope is comprised of a membrane protein and an envelope protein, which are involved in virus assembly, and a spike protein, which mediates entry into host cells. Spike proteins form large trimeric projections, providing the hallmark crown-like appearance of coronaviruses. Spike protein trimers bind to a host receptor and, after priming by cellular proteases, mediate host-virus membrane fusion [6]. These spike proteins appear to be central to viral infectivity by SARS-CoV-2 [6]. The SARS-CoV-2 spike protein binds the host receptor angiotensin converting enzyme 2 (ACE2) with high affinity, and can in cell assays and animal models utilize ACE2 as a functional receptor for host cell entry [7–9].

Casirivimab and imdevimab are two fully human, non-competing, high affinity, immunoglobulin G1 monoclonal antibodies (mAbs) that bind different epitopes on the receptor binding domain of the SARS-CoV-2 spike protein, blocking interaction with the host receptor ACE2 and viral entry into host cells. The casirivimab plus imdevimab (CAS + IMD) combination therapy, administered intravenously (IV) or subcutaneously (SC), has been approved or authorized in several countries for the treatment and prevention of SARS-CoV-2 infection. While it is no longer authorized in the US as the currently dominant variants (Omicron-lineage) are not thought to be susceptible to CAS + IMD [10], next generation anti-SARS-CoV-2 monoclonal antibodies are in development to address ongoing unmet needs, and so understanding the safety, efficacy and pharmacology of existing treatments may help inform development of future therapies, including monoclonal antibodies for other infectious diseases.

The objectives of this population pharmacokinetics (PopPK) analysis, using pooled clinical study data, were to: simultaneously characterize concentration–time profiles of casirivimab and imdevimab, and estimate individual and PopPK parameters of the two mAbs; estimate variability in PK parameters of casirivimab and imdevimab; investigate selected covariates as potential sources of variability in these parameters; and inform dose selection in pediatric population.

## Methods

### Study Population

The dataset used to construct the PopPK model were pooled from seven CAS + IMD clinical studies (Supplemental Tables S1 and S2: ClinicalTrials.gov identifiers: NCT04426695, NCT04425629, NCT04452318, NCT04519437, NCT04666441, NCT05092581, and NCT04992273) and comprised of pediatric and adult non-infected individuals, pediatric and adult ambulatory or hospitalized patients infected with SARS-CoV-2, or household contacts of patients infected with SARS-CoV-2. In these clinical studies, CAS + IMD was administered IV (300 mg to 8000 mg, as a single dose) or SC (600 mg to 1200 mg, as a single dose or 1200 mg every 4 weeks). The PopPK analysis dataset included all individuals who received any amount of study drug and who had at least one non-missing casirivimab or imdevimab measurement following the first dose of study drug.

All studies were conducted in accordance with ethical principles originating from the Declaration of Helsinki and were consistent with the International Conference on Harmonisation/Good Clinical Practices and applicable regulatory requirements. All participants provided written informed consent to be involved in the studies.

### Bioanalytical Assays

Serum samples were analyzed for total casirivimab and total imdevimab using electrochemiluminescent (ECL) immunoassays [11]. The assay procedures employed streptavidin microplates coated with either biotinylated mouse anti-casirivimab mAb, or biotinylated mouse anti-imdevimab mAb. Casirivimab and imdevimab captured on plates specific for each molecule were detected using two ruthenylated, non-competing mouse mAbs, specific to either casirivimab or imdevimab. An ECL signal was generated by the ruthenium label when voltage was applied to the plate using the Meso Scale Discovery reader. The measured electrochemiluminescence (i.e., counts) was proportional to the concentration of total casirivimab or total imdevimab in the serum samples. The lower limit of quantitation (LLOQ) was 0.156 mg/L for both mAbs.

### Modeling Software

Nonlinear mixed effects modeling methodology was implemented in the PopPK analysis using NONMEM® (version 7.5.0, ICON Development Solutions, Ellicott City, MD, USA). The first-order conditional estimation with INTERACTION method (FOCEI) was utilized. A pooled NONMEM-ready data set was constructed using SAS (version 9.4 or later; SAS Institute, Cary, NC, USA) (see Supplemental Material for NONMEM code).

Pre- and post-processing of data from each modeling step and graphical analysis of the data was performed using R (version 4.0.2 or later). Simulations were performed using the R package mrgsolve (0.10.4 or later; Metrum Research Group, Tariffville, CT, USA).

### Data Exclusion and Handling of Records that were Below the Level of Quantification

Participants who received placebo or who had no measurable PK sample following CAS + IMD administration were excluded from the analysis. Samples that were below the level of quantification (BLQ) were excluded given the low frequency of BLQ samples (716 [2.99%] of casirivimab samples and 942 [3.94%] of imdevimab samples). The M3 method [12] was also implemented to account for BLQ censoring during model development and the results supported exclusion of BLQ samples.

### Missing Data and Imputation

If baseline covariates were missing, covariate values collected at another pre-treatment visit were used. If baseline covariates could not be derived this way and only a small number (≤ 10%) of baseline covariate values were missing for a study population, then missing values were imputed at the median value of the study population for continuous covariates or mode for categorical covariates. For time-varying covariates, missing values were imputed using the last observation carry forward rule.

### PopPK Model Building

Base structural model development began with the evaluation of a two-compartment model with first-order absorption following SC administration and first-order elimination for both casirivimab and imdevimab. Concentration data for casirivimab and imdevimab were fitted simultaneously. Different structural and error models were evaluated. Interindividual variability (IIV) was incorporated using a lognormal random effects model on clearance (CL), central volume of distribution (Vc) and absorption rate constant (KA) for both casirivimab and imdevimab, with the same η estimated for casirivimab and imdevimab on CL, KA and Vc (i.e., same η estimated for CL of casirivimab and CL for imdevimab).

$$\theta_i=\theta_{TV}\cdot exp\;\left(\eta_i\right)$$

where θ_i_ is the individual value of the parameter (e.g., CL), θ_TV_ is the typical value model parameter, and η_i_ denotes the interindividual random effect accounting for the ith individual’s deviation from the typical value. The ηi values were assumed to have a normal distribution with zero mean and variance ω^2^.

Residual variability, a composite measure of assay error, dose/sample time collection errors, model misspecification, and any other unexplained variability within a subject, was modeled using an additive error model with log-transformed data for both casirivimab and imdevimab. Separate residual variability terms were estimated for casirivimab and imdevimab.

$$In\;\left(Y_{ij}\right)\;=\;In\;\left(C_{ij}\right)\;+\;e_{ij}$$

where Y_ij_ denotes the observed concentration for the ith individual at time t_j_, C_ij_ denotes the corresponding predicted concentration based on the PK model, and e_ij_ denotes the residual random variability, which was assumed to have a normal distribution with zero mean and variance σ^2^.

A specific covariate, such as baseline body weight, was evaluated at the base structural model building stage to see if it stabilized the model or facilitated identification of the full model in subsequent steps. The base structural model was selected based on successful minimization and completion of covariance steps in NONMEM, precision of parameter estimates, assessment of diagnostic plots, and objective function values (OFV).

The base structural model was used for covariate model building. Covariates were assessed using a full model approach [13] by including all pre-specified covariates on CL and Vc of casirivimab and imdevimab simultaneously, followed by a stepwise backward elimination procedure (change in OFV equivalent to *P* < 0.001) to generate a final model. Covariates evaluated as part of the full model included baseline demographic parameters (age, sex, and race), time-varying serum albumin, baseline hepatic impairment category (according to National Cancer Institute Organ Dysfunction Working Group criteria) [14], baseline viral load, baseline serostatus, baseline disease severity (defined by level of baseline oxygen supplementation therapy required), selected inflammatory biomarkers (including C-reactive protein [CRP], interleukin-8 [IL-8] and neutrophil–lymphocyte ratio [NLR]), which were evaluated as time-varying covariates using data from trials where the inflammatory biomarker information was collected. Correlations between covariates were examined prior to covariate model building to ensure highly correlated (|Pearson correlation coefficient|> 0.6) covariates were not evaluated on the same PK parameter. Clinical judgment and mechanistic plausibility were used to determine which covariates should be tested and on which model parameters. Pregnancy status at baseline was evaluated as an additional covariate following the primary covariate analysis, using the final model as the reference model and forward selection procedure with the same selection criteria used in the backward elimination procedure.

### PopPK Model Evaluation

Model evaluation was performed by examination of a full battery of diagnostic plots, including concordance (e.g., predicted data [PRED] *vs* observed data [DV] and individual predicted data [IPRED] *vs* DV), residual (e.g., conditional weighted residuals [CWRES] *vs* PRED, CWRES *vs* time, individual weighted residuals [IWRES] *vs* IPRED, and IWRES *vs* time), and overlay plots (e.g., DV, PRED, and IPRED *vs* time). A prediction-corrected visual predictive check (pcVPC), stratified by route of administration, was performed to evaluate predictive performance of the final PopPK model.

The impact of individual covariates on the casirivimab and imdevimab area under the curve for a 28-day interval (AUC_day28_) and the concentration 28 days after dosing (C_day28_) following a single dose was assessed visually using forest plots under the final PopPK model.

### PopPK Simulations to Inform Dose Selection for Pediatric Patients

Pediatric doses studied in the clinical trials (Supplemental Table S2) were proposed based on simulations from the PopPK model developed using adult data only, with allometrically scaled clearance and volume of distribution (results not presented in this manuscript). Following the availability of pediatric data from the clinical trials, and the development of the final PopPK model that included pediatric data as reported here, the recommendations for pediatric doses were updated based on simulations from the final PopPK model. The final PopPK model was used to perform simulations to predict casirivimab and imdevimab exposures for adult and pediatric patients infected with SARS-CoV-2 receiving CAS + IMD in order to inform dose recommendations in pediatric patients based on similar exposures of casirivimab and imdevimab as compared to the exposures in adults receiving the approved dose of 1200 mg IV or SC.

A total of 500 virtual patients with normal weight distribution for each weight group were generated by sampling complete covariate vectors for subjects in the observed dataset. A single dose of CAS + IMD was simulated at the dose levels outlined below for each weight group, which were proposed to yield comparable exposures for adults and pediatric patients across different weight groups.

• Adult patients: Single dose of 1200 mg SC or single dose of 1200 mg IV
• Pediatric patients ≥ 20 kg to < 40 kg: Single dose of 480 mg SC or single dose of 650 mg IV
• Pediatric patients ≥ 10 kg to < 20 kg: Single dose of 200 mg SC or single dose of 250 mg IV
• Pediatric patients ≥ 5 kg to < 10 kg: Single dose of 140 mg SC or single dose of 150 mg IV.

## Results

### Analysis Set

After applying pre-defined exclusion rules, the PopPK analysis dataset included 7598 individuals with at least one post-dose observation above the LLOQ and 46,193 (96.5%) quantifiable concentration records (23,210 casirivimab concentration records and 22,983 imdevimab concentration records) from the seven clinical trials.

### Baseline Characteristics of the Study Population

Baseline covariates used for evaluating covariate effects are presented in Table 1. The majority of individuals were adults. Most of the study participants were White and there were approximately equal numbers of males and females. As expected, male participants had higher baseline body weight compared to female participants. The inflammatory biomarkers including CRP and NLR were negatively correlated with albumin.

**Table 1:** Summary Statistics of Covariates and Baseline Demographics

	**Clinical trial**							
	**NCT04426695**	**NCT04425629**	**NCT04452318**	**NCT04519437**	**NCT05092581**	**NCT04992273**	**NCT04666441**	**Total**
**Sex, n (%)**								
Female	579 (44.4)	2252 (52.3)	145 (50.5)	326 (45.0)	1 (100)	5 (71.4)	497 (51.4)	3805 (50.1)
Male	724 (55.6)	2057 (47.7)	142 (49.5)	398 (55.0)	0 (0)	2 (28.6)	470 (48.6)	3793 (49.9)
**Race, n (%)**								
White	835 (64.1)	3702 (85.9)	220 (76.7)	629 (86.9)	1 (100)	6 (85.7)	822 (85.0)	6215 (81.8)
Black or African American	175 (13.4)	216 (5.0)	33 (11.5)	71 (9.8)	0 (0)	1 (14.3)	56 (5.8)	552 (7.3)
Asian	46 (3.5)	132 (3.1)	11 (3.8)	12 (1.7)	0 (0)	0 (0)	51 (5.3)	252 (3.3)
American Indian or Alaska Native	24 (1.8)	51 (1.2)	1 (0.3)	8 (1.1)	0 (0)	0 (0)	5 (0.5)	89 (1.2)
Native Hawaiian or Other Pacific Islander	5 (0.4)	5 (0.1)	2 (0.7)	2 (0.3)	0 (0)	0 (0)	3 (0.3)	17 (0.2)
Not reported	148 (11.4)	83 (1.9)	15 (5.2)	0 (0)	0 (0)	0 (0)	22 (2.3)	268 (3.5)
Unknown	70 (5.4)	120 (2.8)	4 (1.4)	2 (0.3)	0 (0)	0 (0)	8 (0.8)	204 (2.7)
Missing	0 (0)	0 (0)	1 (0.3)	0 (0)	0 (0)	0 (0)	0 (0)	1 (0.0)
**Liver function category, n (%)**								
Normal	721 (55.3)	2940 (68.2)	227 (79.1)	632 (87.3)	1 (100)	2 (28.6)	778 (80.5)	5301 (69.8)
Mild	483 (37.1)	763 (17.7)	36 (12.5)	60 (8.3)	0 (0)	1 (14.3)	70 (7.2)	1413 (18.6)
Moderate	10 (0.8)	12 (0.3)	1 (0.3)	0 (0)	0 (0)	0 (0)	2 (0.2)	25 (0.3)
Severe	8 (0.6)	0 (0)	0 (0)	0 (0)	0 (0)	0 (0)	0 (0)	8 (0.1)
Missing	81 (6.2)	594 (13.8)	23 (8.0)	32 (4.4)	0 (0)	4 (57.1)	117 (12.1)	851 (11.2)
**Oxygen requirement, n (%)**								
No supplemental oxygen	340 (26.1)	4297 (99.7)	287 (100)	0 (0)	0 (0)	0 (0)	0 (0)	4924 (64.8)
Low flow oxygen supply	836 (64.2)	11 (0.3)	0 (0)	0 (0)	1 (100)	0 (0)	958 (99.1)	1806 (23.8)
High flow oxygen supply	104 (8.0)	0 (0)	0 (0)	0 (0)	0 (0)	0 (0)	0 (0)	104 (1.4)
Mechanical ventilation	23 (1.8)	1 (0.0)	0 (0)	0 (0)	0 (0)	0 (0)	0 (0)	24 (0.3)
Missing	0 (0)	0 (0)	0 (0)	724 (100)	0 (0)	7 (100)	9 (0.9)	740 (9.7)
**Age category, n (%)**								
≥ 65 years	545 (41.8)	421 (9.8)	18 (6.3)	90 (12.4)	0 (0)	0 (0)	2 (0.2)	1076 (14.2)
≥ 18 to < 65 years	758 (58.2)	3743 (86.9)	197 (68.6)	634 (87.6)	0 (0)	0 (0)	965 (99.8)	6297 (82.9)
≥ 12 to < 18 years	0 (0)	76 (1.8)	72 (25.1)	0 (0)	0 (0)	0 (0)	0 (0)	148 (1.9)
≥ 9 to < 12 years	0 (0)	42 (1.0)	0 (0)	0 (0)	0 (0)	1 (14.3)	0 (0)	43 (0.6)
≥ 5 to < 9 years	0 (0)	19 (0.4)	0 (0)	0 (0)	0 (0)	2 (28.6)	0 (0)	21 (0.3)
≥ 2 to < 5 years	0 (0)	6 (0.1)	0 (0)	0 (0)	0 (0)	3 (42.9)	0 (0)	9 (0.1)
≥ 1 to < 2 years	0 (0)	1 (0.0)	0 (0)	0 (0)	1 (100)	1 (14.3)	0 (0)	3 (0.0)
< 1 years	0 (0)	1 (0.0)	0 (0)	0 (0)	0 (0)	0 (0)	0 (0)	1 (0.0)
**Age (years)**								
Mean (SD)	60.8 (15.4)	43.6 (16.0)	35.7 (19.0)	47.3 (14.2)	1.00 (NA)	4.86 (3.53)	33.8 (9.65)	45.3 (17.2)
Median [min, max]	61.0 [20.0, 98.0]	44.0 [0, 96.0]	33.0 [12.0, 87.0]	48.0 [18.0, 80.0]	1.00 [1.00, 1.00]	4.00 [1.00, 11.0]	33.0 [18.0, 83.0]	45.0 [0, 98.0]
**Baseline body weight (kg)**								
Mean (SD)	91.2 (25.3)	85.1 (22.5)	79.8 (21.4)	86.3 (21.0)	9.60 (NA)	18.8 (6.55)	73.3 (13.2)	84.5 (22.5)
Median [min, max]	86.6 [38.5, 218]	83.1 [8.60, 235]	77.1 [32.7, 171]	83.4 [35.9, 178]	9.60 [9.60, 9.60]	16.4 [12.5, 31.1]	72.6 [40.0, 121]	81.6 [8.60, 235]
**Baseline BMI (kg/m**^**2**^**)**								
Mean (SD)	31.9 (8.14)	29.8 (6.87)	28.1 (6.70)	29.4 (6.29)	20.2 (NA)	16.5 (1.32)	25.2 (3.35)	29.4 (6.98)
Median [min, max]	30.3 [4.20, 73.0]	29.2 [12.2, 74.2]	26.9 [14.7, 54.0]	28.4 [11.3, 52.8]	20.2 [20.2, 20.2]	16.8 [14.0, 18.0]	25.2 [15.7, 40.2]	28.4 [4.20, 74.2]
**Baseline creatinine clearance (mL/min)**								
Mean (SD)	109 (60.4)	144 (53.2)	145 (50.4)	128 (42.4)	253 (NA)	184 (110)	109 (24.0)	132 (53.1)
Median [min, max]	101 [3.88, 486]	135 [8.15, 487]	138 [37.9, 361]	122 [43.3, 466]	253 [253, 253]	127 [114, 311]	107 [41.7, 226]	124 [3.88, 487]
Missing, n (%)	0 (0)	1 (0.0)	0 (0)	0 (0)	0 (0)	4 (57.1)	0 (0)	5 (0.1)
**Baseline albumin (g/L)**								
Mean (SD)	33.2 (5.52)	42.8 (3.40)	44.3 (2.94)	43.7 (2.64)	40.0 (NA)	45.0 (3.00)	45.4 (3.34)	41.6 (5.46)
Median [min, max]	33.0 [11.0, 80.0]	43.0 [26.0, 56.0]	44.0 [36.0, 54.0]	44.0 [31.0, 53.0]	40.0 [40.0, 40.0]	45.0 [42.0, 48.0]	45.0 [31.0, 56.0]	43.0 [11.0, 80.0]
Missing, n (%)	0 (0)	0 (0)	0 (0)	0 (0)	0 (0)	4 (57.1)	0 (0)	4 (0.1)
**Baseline viral load (log**_**10**_** copies/mL)**								
Mean (SD)	6.01 (2.03)	5.98 (2.49)	0.734 (2.06)	3.97 (1.28)	7.85 (NA)	NA (NA)	6.06 (2.67)	5.78 (2.63)
Median [min, max]	6.27 [0, 10.5]	6.57 [0, 10.5]	0 [0, 8.99]	3.79 [2.55, 5.56]	7.85 [7.85, 7.85]	NA [NA, NA]	6.63 [0, 10.4]	6.40 [0, 10.5]
Missing, n (%)	86 (6.6)	120 (2.8)	22 (7.7)	718 (99.2)	0 (0)	7 (100)	8 (0.8)	961 (12.6)
**Baseline serostatus, n (%)**								
Positive	668 (51.3)	1301 (30.2)	62 (21.6)	77 (10.6)	0 (0)	0 (0)	245 (25.3)	2353 (31.0)
Negative	536 (41.1)	2760 (64.1)	203 (70.7)	612 (84.5)	0 (0)	0 (0)	660 (68.3)	4771 (62.8)
Other	99 (7.6)	248 (5.8)	22 (7.7)	35 (4.8)	0 (0)	0 (0)	62 (6.4)	466 (6.1)
Missing	0 (0)	0 (0)	0 (0)	0 (0)	1 (100)	7 (100)	0 (0)	8 (0.1)
**C-reactive protein (mg/L)**								
Mean (SD)	81.1 (84.2)	10.3 (21.6)	NA (NA)	NA (NA)	NA (NA)	NA (NA)	NA (NA)	26.7 (53.9)
Median [min, max]	61.0 [0, 1410]	3.93 [0.100, 354]	NA [NA, NA]	NA [NA, NA]	NA [NA, NA]	NA [NA, NA]	NA [NA, NA]	5.48 [0, 1410]
Missing, n (%)	0 (0)	0 (0)	287 (100)	724 (100)	1 (100)	7 (100)	967 (100)	1986 (26.1)
**Interleukin-8 (pg/mL)**								
Mean (SD)	38.6 (56.8)	NA (NA)	NA (NA)	NA (NA)	NA (NA)	NA (NA)	NA (NA)	38.6 (56.8)
Median [min, max]	29.3 [4.26, 1260]	NA [NA, NA]	NA [NA, NA]	NA [NA, NA]	NA [NA, NA]	NA [NA, NA]	NA [NA, NA]	29.3 [4.26, 1260]
Missing, n (%)	242 (18.6)	4309 (100)	287 (100)	724 (100)	1 (100)	7 (100)	967 (100)	6537 (86.0)
**Neutrophil/lymphocyte ratio**								
Mean (SD)	8.50 (9.80)	2.41 (1.79)	2.16 (0.866)	2.10 (0.871)	0.820 (NA)	0.740 (0.212)	2.22 (1.54)	3.24 (4.60)
Median [min, max]	5.78 [0.0900, 97.9]	1.96 [0, 27.6]	2.07 [0.590, 5.24]	1.96 [0.570, 7.78]	0.820 [0.820, 0.820]	0.740 [0.590, 0.890]	1.87 [0.160, 13.7]	2.11 [0, 97.9]
Missing, n (%)	346 (26.6)	561 (13.0)	8 (2.8)	0 (0)	0 (0)	5 (71.4)	163 (16.9)	1083 (14.3)

*BMI* Body mass index, *NA* Not available, *SD* Standard deviation

### Base Model

The base structural PK model for both casirivimab and imdevimab was a two-compartment model with first-order absorption following SC dose administration and first-order elimination, with simultaneous fitting of casirivimab and imdevimab concentration data. Base structural model development started with estimating separate sets of PK parameters CL, Vc, Vp (peripheral volume of distribution), intercompartmental clearance (Q), KA and bioavailability (F1) for casirivimab and imdevimab. Different structural models with shared PK parameters among casirivimab and imdevimab were tested, and all resulted in increased OFV values. Therefore, the final base structural model had separate PK parameters estimated for casirivimab and imdevimab. IIV random effects were included on CL, Vc and KA for both casirivimab and imdevimab, with the same η estimated for casirivimab and imdevimab on CL, Vc and KA (i.e., same η estimated for CL of casirivimab and CL for imdevimab). The effect of baseline body weight on CL and Vc for casirivimab and imdevimab was included as a structural covariate in the base structural model with the shared exponents estimated for casirivimab and imdevimab (i.e., same exponent estimated for the body weight effect on CL for both casirivimab and imdevimab).

To evaluate the influence of BLQ samples, the base structural model was run with BLQ samples included, using the M3 method, and the estimates of the model parameters were compared to the estimates from the base structural model with BLQ samples excluded. The differences in key parameters (e.g., CL, Vc) were < 10%; therefore, the BLQ samples were considered unlikely to influence casirivimab and imdevimab exposures and were excluded from the subsequent model development steps, which was expected given the low frequency (< 5%) of the BLQ samples.

### Covariate Model

The covariate model was developed using a full model approach, where the following non-structural covariates were added to the CL and Vc of both casirivimab and imdevimab simultaneously: baseline age, time-varying serum albumin, sex, race, baseline hepatic impairment status, baseline viral load, baseline serostatus, level of oxygen supplementation therapy at baseline, CRP, IL-8, and NLR. The full model was reduced to a working full model by removing covariate effects with poor precision (percentage relative standard error [%RSE] > 100%): age, race, baseline viral load, IL-8 and NLR on Vc for both casirivimab and imdevimab. The working full model was stable with a 11.9 unit increase in OFV value as compared to the full model with 10 less parameters.

Stepwise backward elimination procedure based on the likelihood ratio test was performed on the working full model to identify a parsimonious final PK model. Eight of the 34 non-structural covariates in the working full model did not meet the inclusion criteria (ΔOFV > 10.8 [*P* < 0.001]) and were removed. Model refinement was conducted following backward elimination to assess if the same covariate effect on CL and Vc for casirivimab and imdevimab could be shared (e.g., same θ estimated for the same covariate effect on CL or Vc for casirivimab and imdevimab). Model refinement results showed < 3 unit increase in OFV value when the same θ was estimated for the following covariate effects for casirivimab and imdevimab: baseline age, race, baseline hepatic impairment status, baseline viral load, baseline serostatus, CRP, NLR, level of oxygen supplementation therapy at baseline on CL, and time-varying albumin on Vc. Thus, the effects of these covariates on CL and Vc of casirivimab and imdevimab were determined to be shared by estimating with the same θ.

Pregnancy status at baseline was evaluated on CL and Vc of casirivimab and imdevimab following the primary covariate analysis. A total of 66 pregnant women were included in the analysis dataset, casirivimab and imdevimab PK parameters did not appear to differ significantly from the overall population in this small subset of women.

### Final Model

As the analysis dataset included pediatrics from birth to < 18 years of age, with higher bioavailability following SC injection expected in young children [15] and model diagnostics suggested larger exponents of body weight on CL and Vc of casirivimab and imdevimab were needed for pediatrics at younger age (mainly < 6 years old), several model refinements were undertaken to improve the fit for children following the covariate model building. The final PK model following the model refinement had a separate bioavailability term estimated for pediatrics and fixed exponents describing body weight effect on CL and Vc to classical allometric exponents (0.75 for CL and 1 for Vc) for children < 6 years of age. Incorporating these changes resulted in discernible improvements in model fitting for pediatrics, especially for children < 6 years old. In addition to the refinements, investigations were made to see if additional maturation changes due to age needed to be accounted for in addition to the body weight effect. An asymptotic exponential maturation function of age was evaluated on CLs for both casirivimab and imdevimab, with the equation for CL defined as follows:

$${CL}_{i}={\theta }_{CL, adult}{\cdot (\frac{{WT}_{i}}{70 kg})}^{\theta WT}\cdot (1-(1-\beta )\cdot \text{exp}(-PNA\cdot \frac{\text{ln}\left(2\right)}{{T}_{CL}}))\cdot \text{exp}({\eta }_{CL})$$

where *CL*_*i*_ is the CL value for the ith individual, ${\theta }_{CL, adult}$ is the population CL for adults, *WT*_*i*_ is the individual body weight in kg normalized by a reference body weight of 70 kg, $\theta WT$ is the power of the normalized body weight covariate on CL, $PNA$ is the postnatal age in years, $\beta$ is the parameter in the maturation function that defines the estimated fraction of adult CL that is present at birth, ${T}_{CL}$ is the maturation half-life for CL in years, and ${\eta }_{CL}$ is the IIV random effect on CL. Incorporating the age maturation function on CLs for casirivimab and imdevimab resulted in unrealistic estimated maturation half-life for CLs with no improvements in model fitting based on diagnostic plots. As the majority (> 98%) of pediatric subjects in the analysis dataset were aged 2 years or older, and age-related maturation effect in addition to the body weight effect is not expected in children > 2 years or in adolescents [16, 17], it was determined that the impact of maturation in pediatric subjects on CLs of casirivimab and imdevimab can be sufficiently explained by body weight alone and that additional incorporation of maturation function in term of age was not necessary.

Covariates included in the final model were: age, sex, race, time-varying serum albumin, mild hepatic impairment at baseline, baseline viral load, baseline serostatus, time-varying CRP, time-varying NLR, and level of oxygen supplementation therapy at baseline on CL, and sex and time-varying albumin on Vc for both casirivimab and imdevimab, in addition to baseline body weight, which was included as a structural covariate on CL and Vc for casirivimab and imdevimab. All of these covariates were independent of each other with |Pearson correlation coefficient|< 0.6. Covariate effects for casirivimab and imdevimab are described in Eqs. 1– 4.

1 $${TVCL}_{i, casirivimab}={{\theta }_{1}\cdot (\frac{{WT}_{i}}{81.6})}^{({\theta }_{13}\cdot \left(1-PED\right)+0.75\cdot PED)}\cdot {\left(\frac{{Age}_{i}}{45}\right)}^{{\theta }_{15}}\cdot \left(1+{\theta }_{16}\cdot SEXF\right)\cdot \left(1+{\theta }_{17}\cdot RACE\right)\cdot {\left(\frac{{ALB}_{i}}{43}\right)}^{{\theta }_{18}}{\cdot \left(1+{\theta }_{19}\cdot HEPIMP\right)\cdot \left(\frac{{VIRAL}_{i}}{6.4}\right)}^{{\theta }_{20}}\cdot \left(1+{\theta }_{21}\cdot SERPOS\right){\cdot \left(\frac{{CRP}_{i}}{5.48}\right)}^{{\theta }_{22}}{\cdot \left(\frac{{NLR}_{i}}{2.11}\right)}^{{\theta }_{24}}\cdot \left(1+{\theta }_{25}\cdot OXYSTAT1\right)\cdot \left(1+{\theta }_{26}\cdot OXYSTAT2\right)$$

2 $${TVCL}_{i, imdevimab}={{\theta }_{5}\cdot (\frac{{WT}_{i}}{81.6})}^{({\theta }_{13}\cdot \left(1-PED\right)+0.75\cdot PED)}\cdot {\left(\frac{{Age}_{i}}{45}\right)}^{{\theta }_{15}}\cdot \left(1+{\theta }_{38}\cdot SEXF\right)\cdot \left(1+{\theta }_{17}\cdot RACE\right){\cdot \left(\frac{{ALB}_{i}}{43}\right)}^{{\theta }_{40}}{\cdot \left(1+{\theta }_{19}\cdot HEPIMP\right)\cdot \left(\frac{{VIRAL}_{i}}{6.4}\right)}^{{\theta }_{20}}\cdot \left(1+{\theta }_{21}\cdot SERPOS\right){\cdot \left(\frac{{CRP}_{i}}{5.48}\right)}^{{\theta }_{22}}{\cdot \left(\frac{{NLR}_{i}}{2.11}\right)}^{{\theta }_{24}}\cdot \left(1+{\theta }_{25}\cdot OXYSTAT1\right)\cdot \left(1+{\theta }_{26}\cdot OXYSTAT2\right)$$

SEXF = 1 if female or = 0 for male;

RACE = 1 for White or = 0 for non-White;

PED = 1 for pediatrics < 6 years of age or = 0 for others;

HEPIMP = 1 for mild hepatic impairment or = 0 for others;

SERPOS = 1 for positive serostatus at baseline or = 0 for negative serostatus at baseline;

OXYSTAT1 = 1 for low oxygen supplement therapy or = 0 for no oxygen supplement therapy;

OXYSTAT2 = 1 for high oxygen supplement therapy or = 0 for no oxygen supplement therapy.

${TVCL}_{i, casirivimab}$ and ${TVCL}_{i, imdevimab}$ denotes the ith individual’s typical value of CL for casirivimab and imdevimab, respectively. ${\theta }_{13,\text{ 15,16}, 17,\text{ 18,19},\text{ 20,21},\text{ 22,24,25,26,38,40}}$ are parameters of the model that describe the influence of the corresponding covariate. ${\theta }_{1}$ and ${\theta }_{5}$ represent casirivimab CL and imdevimab CL of a hypothetical 45-year-old, non-White male subject weighing 81.6 kg with an albumin of 43 g/L, baseline viral load of 6.4 log_10_ copies/mL, CRP of 5.48 mg/L, NLR of 2.11, negative serostatus, and not on oxygen supplementation therapy.

3 $${TVVc}_{i, casirivimab}={{\theta }_{2}\cdot (\frac{{WT}_{i}}{81.6})}^{{(\theta }_{14}\cdot \left(1-PED\right)+1\cdot PED)}\cdot \left(1+{\theta }_{28}\cdot SEXF\right){\cdot \left(\frac{{ALB}_{i}}{43}\right)}^{{\theta }_{30}}$$

4 $${TVVc}_{i, imdevimab}={{\theta }_{6}\cdot (\frac{{WT}_{i}}{81.6})}^{{(\theta }_{14}\cdot \left(1-PED\right)+1\cdot PED)}\cdot \left(1+{\theta }_{50}\cdot SEXF\right){\cdot \left(\frac{{ALB}_{i}}{43}\right)}^{{\theta }_{30}}$$

SEXF = 1 if female or = 0 for male.

${TVVc}_{i,casirivimab}$ and ${TVVc}_{i,imdevimab}$ denotes the ith individual’s typical value of Vc for casirivimab and imdevimab, and ${\theta }_{14, \text{28,30,50}}$ are parameters of the model that describe the influence of the corresponding covariate. ${\theta }_{2}$ and ${\theta }_{6}$ represents casirivimab Vc and imdevimab Vc of a hypothetical male subject weighing 81.6 kg with an albumin of 43 g/L.

Estimated values for the final PopPK model parameters of CL, Vc, Vp, KA and Q for casirivimab for a typical 45-year-old, non-White male subject weighing 81.6 kg with a albumin of 43 g/L, baseline viral load of 6.4 log_10_ copies/mL, CRP of 5.48 mg/L, NLR of 2.11, negative serostatus, and not on oxygen supplementation therapy were 0.193 L/day, 3.917 L, 3.065 L, 0.218 day^–1^ and 0.413 L/day (Table 2). Estimated values for the final PopPK model parameters of CL, Vc, Vp, KA and Q for imdevimab for a typical subject with the same conditions as described above were 0.236 L/day, 3.823 L, 3.200 L, 0.235 day^–1^ and 0.403 L/day (Table 2). The bioavailability of casirivimab and imdevimab following SC administration were estimated to be 72.0% and 66.2% for adults and 87.9% and 84.0% for pediatrics, respectively. All PK parameters were well estimated with %RSE 7% or less for structural parameters and 30% or less for covariate effects (Table 2). Estimates of IIV (as % coefficient of variation) for CL, Vc and KA of casirivimab and imdevimab were 30.0%, 34.6% and 78.3%, respectively. The η shrinkage were low for CL, Vc and KA (19.6%, 13.6% and 20.6%, respectively).

**Table 2:** Population Pharmacokinetic Parameter Estimates and 95% Confidence Intervals for the Final Model of Casirivimab and Imdevimab

Parameter (units)	Estimate		%RSE		95% CI	
	**Casirivimab**	**Imdevimab**	**Casirivimab**	**Imdevimab**	**Casirivimab**	**Imdevimab**
CL: Clearance (L/day)	0.1926	0.2359	1.517	1.533	(0.1869, 0.1983)	(0.2288, 0.243)
Vc: Central volume of distribution (L)	3.9170	3.823	1.033	1.025	(3.838, 3.996)	(3.746, 3.9)
Q: Intercompartmental clearance (L/day)	0.4131	0.4029	5.573	4.604	(0.368, 0.4582)	(0.3665, 0.4393)
Vp: Peripheral volume of distribution (L)	3.065	3.199	1.879	1.771	(2.952, 3.178)	(3.088, 3.31)
KA: Absorption rate constant (1/day)	0.2183	0.2354	6.619	6.648	(0.19, 0.2466)	(0.2047, 0.2661)
Bioavailability	0.72	0.6617	1.172	1.208	(0.7035, 0.7365)	(0.646, 0.6774)
Weight on CL	0.7959		2.577		(0.7557, 0.8361)	
Weight on Vc	0.5392		4.457		(0.4921, 0.5863)	
Age on CL	0.07037		18.500		(0.04485, 0.09589)	
Sex on CL	–0.08051	–0.06919	11.33	13.30	(–0.09839, –0.06263)	(–0.08723, –0.05115)
Race on CL	–0.09478		12.72		(-0.1184, -0.07114)	
Albumin on CL	–1.078	–0.9643	4.762	5.241	(–1.179, –0.9774)	(–1.063, –0.8652)
Hepatic impairment on CL	0.06602		19.12		(0.04128, 0.09076)	
Viral load on CL	–0.00754		25.68		(–0.01133, –0.003745)	
Serostatus on CL	0.07315		15.69		(0.05065, 0.09565)	
CRP on CL	0.02252		22.60		(0.01254, 0.0325)	
NLR on CL	0.02883		29.13		(0.01237, 0.04529)	
Low oxygen supply on CL	0.1064		13.09		(0.0791, 0.1337)	
High oxygen supply on CL	0.3802		15.24		(0.2666, 0.4938)	
Sex on Vc	–0.1092	–0.08783	12.24	16.13	(–0.1354, –0.08299)	(–0.1156, –0.06006)
Albumin on Vc	–0.4167		9.225		(–0.492, –0.3414)	
Bioavailability in pediatrics	0.8788	0.8401	4.716	4.717	(0.7976,0.96)	(0.7624,0.9178)
Residual variability	23.52	23.72	4.571	3.952	(23.51, 23.53)	(23.71, 23.73)
IIV in CL (%CV)	30.04		2.124		(30.03, 30.05)	
IIV in Vc (%CV)	34.58		5.888		(34.55, 34.61)	
IIV in KA (%CV)	72.28		9.354		(78.05, 78.51)	
Objective function	–56426.6					

Albumin, CRP and NLR were evaluated as time-varying covariates, all other covariates were stationary

*CI* Confidence interval, *CRP* C-reactive protein, *CV* Coefficient of variation, *IIV* Inter-individual variability, *NLR* Neutrophil–lymphocyte ratio, *RSE* Relative standard error

**Fig. 1:**
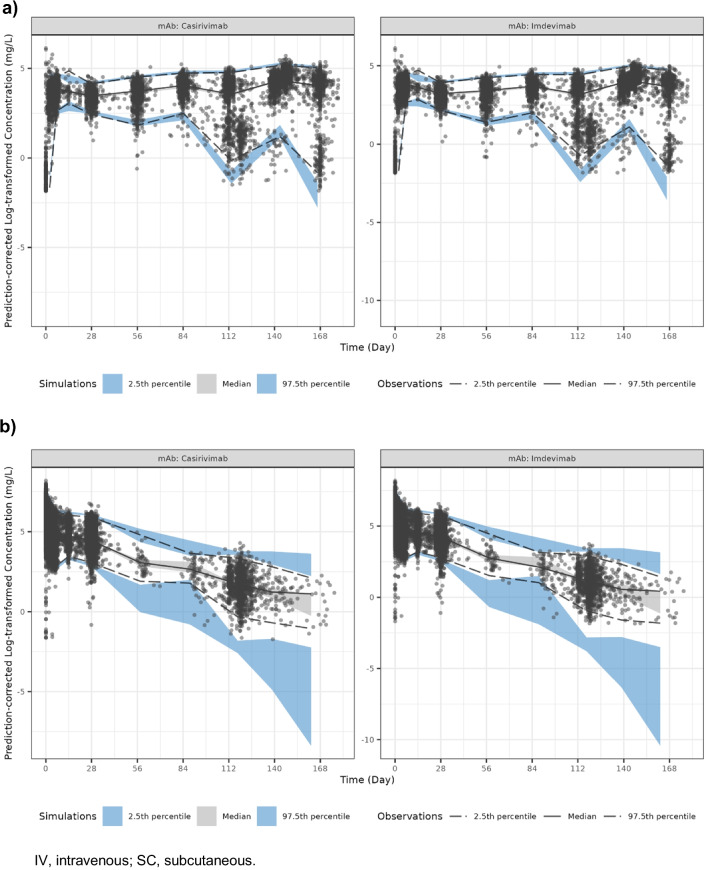
Prediction-corrected visual predictive check plots for the final population pharmacokinetics model: (**a**) SC route and (**b**) IV route

Inspection of the diagnostic plots (Supplemental Fig. S1 [log-transformed data] and Supplemental Fig. S2 [back-transformed data]) suggests good agreement between the observed data and the model predictions without any trends indicating obvious bias. No unacceptable trends were observed in plots of CWRES *vs* PRED or CWRES *vs* TIME, suggesting appropriate residual error structure was implemented. Within the dataset, 43 observations were identified as potential outliers, defined as |CWRES|> 10. The influence of these outliers was evaluated by running the final PK model with these outliers excluded and estimates of the model parameters were compared to the estimates from the final PK model (with outliers included). The differences in key PK parameter were < 7%, therefore the outliers were not considered influential and were retained in the final model. The predictive performance of the final PK model was evaluated through a pcVPC (Fig. 1 [log-transformed data], Supplemental Fig S3 [back-transformed data on log_10_ scale], and Supplemental Fig S3 [based on time after dose]). The pcVPC results demonstrated good consistency in central tendency between simulated casirivimab and imdevimab concentration data and the observed data, with over-predicted variability observed at low concentrations (i.e., < 1 mg/L), suggesting good qualification of the final PK model and reliable predictions for individual exposures above the concentration range of approximately 1 mg/L.

**Fig. 2:**
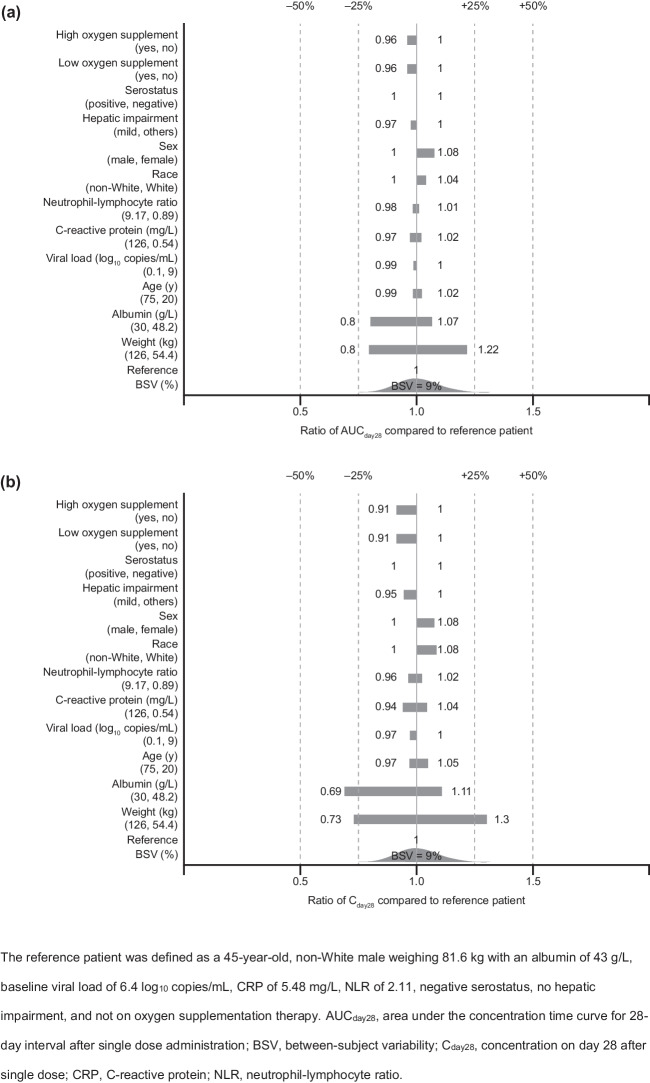
Forest plots showing the effect of statistically significant covariates on casirivimab exposures: (**a**) AUC_day28_ and (**b**) C_day28_

**Fig. 3:**
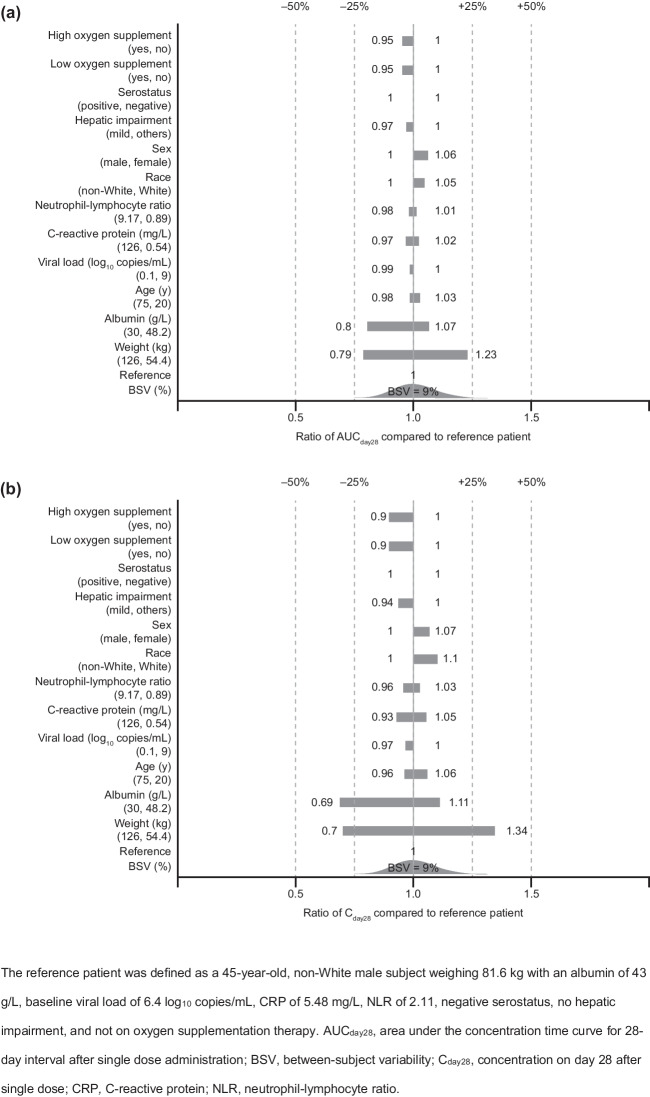
Forest plots showing the effect of statistically significant covariates on imdevimab exposures: (**a**) AUC_day28_ and (**b**) C_day28_

The impact of individual statistically significant covariates on derived PK exposures is illustrated in the forest plots in Fig. 2 for casirivimab and Fig. 3 for imdevimab. Baseline body weight demonstrated the largest predicted effect on casirivimab and imdevimab exposures, such that the predicted exposures (AUC_day28_ and C_day28_) for a subject with extreme baseline body weight (5th and 95th percentile) deviated from a typical subject by 20% to 30% for casirivimab and 21% to 34% for imdevimab. Albumin was predicted to have approximately 7% to 31% impact on casirivimab and imdevimab exposures at the 5th and 95th percentiles of albumin values in the study population compared to the median. Other covariate effects were all predicted to have a small (< 10% change in exposures) impact on casirivimab and imdevimab exposures.

### Simulations to Inform Dose Selection for Pediatrics

**Fig. 4:**
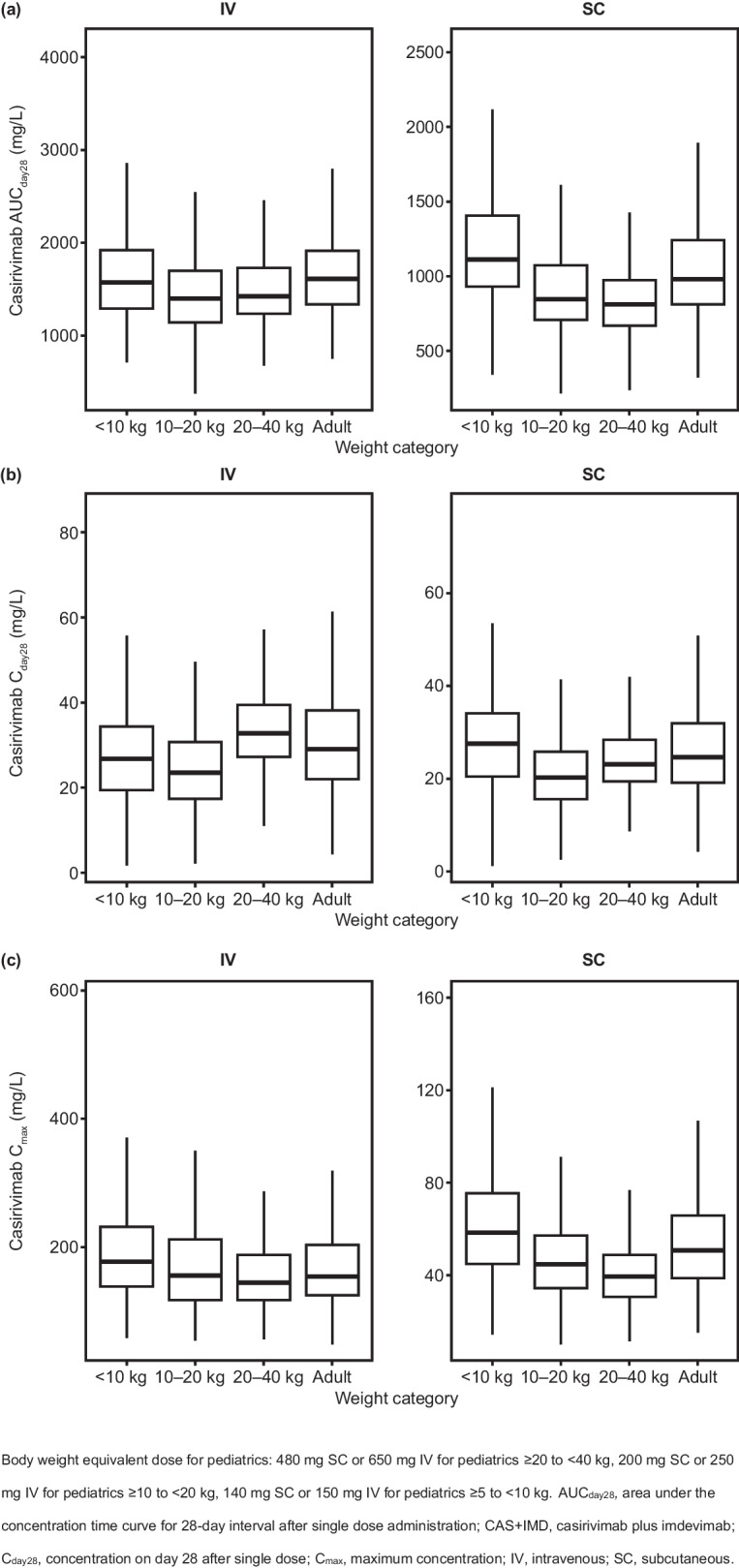
Simulated casirivimab exposures following single IV or SC dose of 1200 mg CAS + IMD or body weight equivalent dose by weight groups: (**a**) AUC_day28_, (**b**) C_day28_ and (**c**) C_max_

**Fig. 5:**
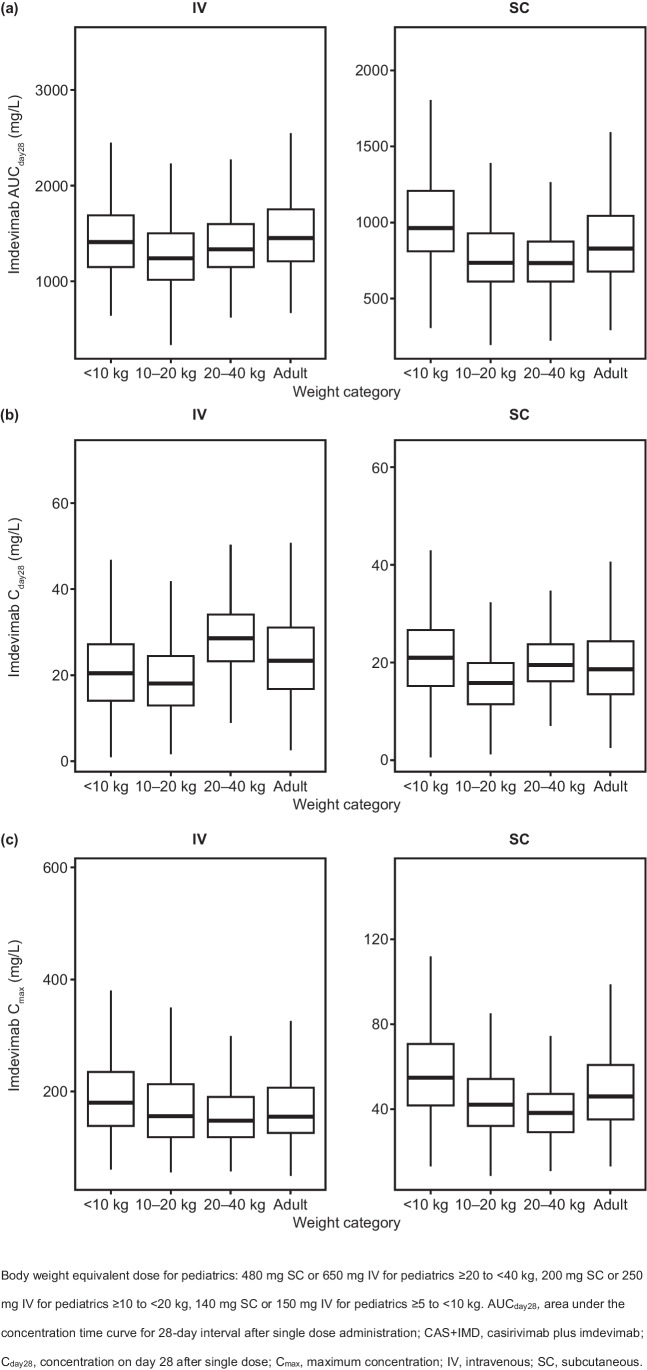
Simulated imdevimab exposures following single IV or SC dose of 1200 mg CAS + IMD or body weight equivalent dose by weight groups: (**a**) AUC_day28_, (**b**) C_day28_ and (**c**) C_max_

Simulations of casirivimab and imdevimab exposures in 500 virtual patients with normal weight distribution for each weight group following a single dose of 1200 mg IV or SC for adults, or dose equivalent for pediatrics, showed similar distribution of exposures in adult and pediatric patients following the proposed weight-based dose as outlined in the Methods section (Figs. 4 and 5).

## Discussion

A PopPK model was developed for casirivimab and imdevimab using concentration–time data from seven clinical studies (two Phase 1/2/3, one Phase 1, one Phase 1b, one Phase 2, one Phase 2a and one Phase 3 studies) in non-infected pediatric and adult individuals, ambulatory or hospitalized pediatric and adult patients infected with SARS-CoV-2, or household contacts of patients infected with SARS-CoV-2 receiving SC or IV CAS + IMD. As indicated by the observed data, concentrations of casirivimab and imdevimab are highly similar, therefore as expected, the concentration–time course of casirivimab and imdevimab can be adequately described by the same structural PK model simultaneously: the two-compartment disposition models with linear absorption following SC administration, direct IV administration into the central compartment, and linear elimination. Based on the observed data, concentrations of casirivimab and imdevimab increased in a dose proportional manner and displayed no evidence of non-linearity in the elimination phase. Baseline body weight was included as a structural covariate on both CL and Vc for casirivimab and imdevimab in the base PK model.

Separate bioavailability terms for both casirivimab and imdevimab were estimated for children in the final PopPK model, where the bioavailability following SC administration was 16% and 18% higher in children compared to adults for casirivimab and imdevimab, respectively. Higher bioavailability in children could be due to increased extracellular fluid volume, higher perfusion rate, and relatively less thickness of SC tissue compared to adults.

Separate PK parameters including CL, Vc, Vp, Q, KA and F1 were estimated for casirivimab and imdevimab. The PK parameter estimates for clearances are 0.193 and 0.236 L/day for casirivimab and imdevimab, respectively. The linear elimination of casirivimab and imdevimab is expected to be non-specific and limited to proteolytic catabolism. This process is relatively slow as the mAbs can be salvaged via binding to the neonatal Fc receptor [18]. The half-life estimated based on PK parameters for casirivimab and imdevimab are 27.6 and 23.5 days, respectively, which are similar to those for a typical endogenous immunoglobulin G1 type antibody [19]. The estimate for central volume of distribution for casirivimab and imdevimab are 3.92 and 3.82 L, respectively, which are close to the plasma volume of approximately 3 L in typical adults. Steady-state volume (Vss = Vc + Vp) estimates for casirivimab and imdevimab are 6.98 and 7.02 L, respectively. The estimates of Vc and Vss are consistent with other mAbs and is an indication that the distribution of casirivimab and imdevimab are mainly restricted to the vascular space, similar to other large protein therapeutics. The IIV for CL and Vc was moderate (approximately 30% and 35%, respectively) and was expected considering linear elimination by non-specific, high-capacity endocytosis and limited space of distribution primarily restricted to vascular compartment.

Given that PK parameters and concentrations of casirivimab and imdevimab are highly similar, as expected, the same covariates were identified to have a statistically significant effect on CL of casirivimab and imdevimab: baseline body weight, age, sex, race, time-varying serum albumin, baseline viral load, time-varying selected inflammatory biomarkers (CRP and NLR), status of hepatic impairment at baseline, baseline serostatus and oxygen supplementation therapy at baseline. Baseline body weight, sex and time-varying albumin were also found to be statistically significant covariates on the Vc for casirivimab and imdevimab. Among the covariate effects included in the final model, baseline body weight had the largest predicted impact on exposures (AUC_day28_ and C_day28_), as is typical for therapeutic antibodies, with patients at the 5th (54.4 kg) and 95th (126 kg) percentiles of body weight predicted to have approximately 22% to 34% higher and 20% to 30% lower exposures than the reference 81.6 kg subject. Consequently, doses for pediatrics need to be adjusted based on body weight regardless of administration route. Simulation results demonstrated that following the weight-based dosing regimens, comparable exposures of casirivimab and imdevimab are expected between different weight groups in pediatrics and adults (Figs. 4 and 5), supporting the assumption that efficacy and safety of CAS + IMD treatment is likely to be similar across pediatric age/body weight groups as compared to adults. CLs for casirivimab and imdevimab were inversely related to albumin, such that an increase in albumin corresponded to a decrease in predicted CLs. This effect may be due to a common pathway of protein catabolism impacting both albumin and casirivimab/imdevimab, such that lower albumin levels reflect increased protein catabolism and thus increased clearances for casirivimab and imdevimab [19, 20]. The magnitude of this effect is modest for both casirivimab and imdevimab, reflecting approximately 7% to 31% change in CL at the extremes of albumin (5th and 95th percentiles) compared to the median. Baseline disease severity as defined by the level of oxygen supplementation therapy required, along with a selected panel of inflammatory biomarkers such as CRP and NLR were identified as statistically significant predictors of increased CLs of casirivimab and imdevimab, which were above and beyond albumin level which was statistically inversely correlated with CLs magnitude. This may be related to physiological changes secondary to an increased inflammatory state in these patients. However, the magnitude of these covariate effects was considered small with 9% lower exposures in patients receiving high oxygen supply and < 5% change in exposures at the 5th and 95th percentiles of the CRP and NLR values in the study population compared to patients not on oxygen supply with median biomarker values. Other intrinsic subject factors included as statistically significant covariates in the final model (age, sex, race, baseline viral load, status of hepatic impairment at baseline, and baseline serostatus) were predicted to have a small impact on exposures of casirivimab and imdevimab. Despite the small effect on casirivimab and imdevimab exposures, these covariates were retained in the final model based on statistical significance criteria and were not expected to have a clinically meaningful impact.

The pcVPC results showed over-predicted variability at low concentrations (i.e., < 1 mg/L). A plausible source for this bias is the lack of dense data at this range of concentrations. As a result, model predictions at this low range of concentrations should be interpreted with caution. It is worth noting however, that the goal of CAS + IMD combination therapy was to maintain concentrations of casirivimab and imdevimab above the neutralization target concentrations for variants susceptible to CAS + IMD (approximately 20 mg/L or higher for each mAb) for 28 days [21], therefore the developed PopPK model was considered suitable in supporting dose recommendation for CAS + IMD combination therapy.

This analysis had several strengths including simultaneous administration of two highly similar mAbs, offering unique insight into the PK of the two mAbs simultaneously utilizing a large and diverse study population pooled across trials. The inclusion of pediatric data allows better characterization of the casirivimab and imdevimab PK in this population, and the developed final PopPK model was used to support weight-based dose recommendations in a pediatric population that are expected to yield comparable exposures to those in adults receiving the approved dose of 1200 mg IV or SC. Meanwhile, our analysis had limitations due to trial designs and operational challenges. One limitation was that only baseline, but not time-varying viral load information, was available and evaluated as a covariate in this analysis. Given that viral load is considered as the drug target, evaluating viral load as a time-varying covariate effect could be indicative that a target-mediated drug disposition effect was not captured due to study design and available data at the time the analysis was performed. Another example was that although the study population included a wide age range (< 1 to 98 years), only a limited amount of young children (< 6 years of age) were included, consisting of around 0.5% of the whole population. Consequently, exponents of body weight on CL and Vc of casirivimab and imdevimab were fixed to classical allometric exponents of 0.75 for CL and 1 for Vc, for children < 6 years of age to better characterize PK profiles of casirivimab and imdevimab in this younger population. It would be pertinent to explore the PK characteristics of casirivimab and imdevimab in a young pediatric population to affirm the influence of body weight and age.

## Conclusion

Concentration–time data of casirivimab and imdevimab collected in seven clinical trials were appropriately described simultaneously by two compartment models with first-order absorption following SC dose administration and first-order elimination. The final PopPK model provided well-estimated parameters for both casirivimab and imdevimab. Among the covariates identified as statistically significant, body weight had the largest impact while all other covariates resulted in small differences on casirivimab and imdevimab exposures, which are unlikely to be influential on dose adjustment. Model predictions suggest comparable exposures of casirivimab and imdevimab are expected between different weight groups in pediatrics and adults following weight-based dosing regimens in pediatrics. Overall, the PopPK analysis provided important PK characteristics of casirivimab and imdevimab in a diverse population including both pediatric and adult non-infected individuals, pediatric and adult ambulatory and hospitalized patients infected with SARS-CoV-2, and informed dose selection in pediatric population aged 2–12 years old weighing at least 10 kg.

## Supplementary Information

Supplementary Material 1.

## Data Availability

Qualified researchers may request access to study documents (including the clinical study report, study protocol with any amendments, blank case report form, and statistical analysis plan) that support the methods and findings reported in this manuscript. Individual anonymized participant data will be considered for sharing once the product and indication has been approved by major health authorities (e.g. Food and Drug Administration, European Medicines Agency, Pharmaceuticals and Medical Devices Agency, etc.), if there is legal authority to share the data and there is not a reasonable likelihood of participant re-identification. Requests should be submitted to https://vivli.org/.
