# Supplementary material for: Population Pharmacokinetics of Casirivimab and Imdevimab in Pediatric and Adult Non-Infected Individuals, Pediatric and Adult Ambulatory or Hospitalized Patients or Household Contacts of Patients Infected with SARS-COV-2
Source: Pharm Res. 2024 Sep 18;41(10):1933–49. doi: 10.1007/s11095-024-03764-5 (PMC11530482; doi:10.1007/s11095-024-03764-5)
Supplement: Supplementary file 1 — Supplementary Material 1. [file 11095_2024_3764_MOESM1_ESM.docx]

# Supplemental Material. NONMEM codes

$SIZES PD=100

$PROBLEM REGN-COV Joint POPPK for R10933 & R10987 IV/SC

$INPUT C ROWID STDY ID ACTARMN TIME TAD NTIM AMT DOSEDUR=DROP RATE ROUTN EVID DVIDN DV DVOR BLQ CMT=DROP CMT MDV PHASE=DROP TRTAN DOSEA SERBLN HRSTATBL IMMCOD SYMBL COVSBL COVSTAT OXYBL AGECTN AGEBL1 AGE SEXN RACEN RACB ETHNICN=DROP WGTBL WGT BMIBL BMI VRQNP1 ALBBL1 ALB1 ASTBL ALTBL CREATBL=DROP CRCLBL BILIBL=DROP LIVRFNCN HEPMILD1 HEPMOD CRPBL CRP1 INT8BL INT81 NLR1 NLRBL IMUNSUPP ORDER ADA33 ADA87 CSBL=DROP PAGEBL PAGE L2=DROP L3=DROP

$DATA ../../DerivedData/nmdat_20230308.csv

IGNORE = @

IGNORE = C$THETA

(0,0.2) ; th1: CL1 - CL for R10933 (L/Day)

(0,4.14) ; th2: V2 - Vc for R10933 (L)

(0,0.3) ; th3: Q1 - Q for R10933 (L/Day)

(0,3.26) ; th4: V3 - Vp for R10933(L)

(0,0.2) ; th5: CL2 - CL for R10987 (L/Day)

(0,4) ; th6: V5 - Vc for R10987 (L)

(0,0.3) ; th7: Q2 - Q for R10987 (L/Day)

(0,3.5) ; th8: V6 - Vp for R10987 (L)

(0,0.2) ; th9: KA1: KA for R10933 (1/Day)

(0,0.2) ; th10: KA2: KA for R10987 (1/Day)

(0,0.7,1) ; th11: F1: F for R10933

(0,0.7,1) ; th12: F4: F for R10987

(0.9) ; th13: Exponent of WT on CL

(0.6) ; th14: Exponent of WT on Vc

(0.1) ; th15: Age on CL1

(-0.1) ; th16: Sex on CL1

(-0.1) ; th17: Race on CL1

(-0.5) ; th18: Albumin on CL1

(0.1) ; th19: Hepatic impairment on CL1

(-0.01) ; th20: Viral load on CL1

(0.1) ; th21: Serostatus on CL1

(0.1) ; th22: CRP on CL1

(0 FIX) ; th23: IL8 on CL1

(0.1) ; th24: NLR on CL1

(0.1) ; th25: Low Oxygen supply on CL1

(0.1) ; th26: High Oxygen supply on CL1

(0 FIX) ; th27: Age on V2

(-0.1) ; th28: Sex on V2

(0 FIX) ; th29: Race on V2

(-0.1) ; th30: Albumin on V2

(0 FIX) ; th31: Hepatic impairment on V2

(0 FIX) ; th32: Viral load on V2

(0 FIX) ; th33: Serostatus on V2

(0 FIX) ; th34: CRP on V2

(0 FIX) ; th35: IL8 on V2

(0 FIX) ; th36: NLR on V2

(0 FIX) ; th37: Age on CL2

(-0.1) ; th38: Sex on CL2

(0 FIX) ; th39: Race on CL2

(-0.1) ; th40: Albumin on CL2

(0 FIX) ; th41: Hepatic impairment on CL2

(0 FIX) ; th42: Viral load on CL2

(0 FIX) ; th43: Serostatus on CL2

(0 FIX) ; th44: CRP on CL2

(0 FIX) ; th45: IL8 on CL2

(0 FIX) ; th46: NLR on CL2

(0 FIX) ; th47: Low Oxygen supply on CL2

(0 FIX) ; th48: High Oxygen supply on CL2

(0 FIX) ; th49: Age on V5

(-0.1) ; th50: Sex on V5

(0 FIX) ; th51: Race on V5

(0 FIX) ; th52: Albumin on V5

(0 FIX) ; th53: Hepatic impairment on V5

(0 FIX) ; th54: Viral load on V5

(0 FIX) ; th55: Serostatus on V5

(0 FIX) ; th56: CRP on V5

(0 FIX) ; th57: IL8 on V5

(0 FIX) ; th58: NLR on V5

(0,0.7) ; th59: F1: F for R10933 (peds)

(0,0.7) ; th60: F4: F for R10987 (peds)

$OMEGA

0.1 ; eta1: IIV in CL

0.1 ; eta2: IIV in Vc

0.1 ; eta3: IIV in KA

$SIGMA

0.5 ; EPS1: RV

0.5 ; EPS2: RV

$SUBROUTINE ADVAN13 TOL=6

$MODEL

COMP = (DEPOT_33) ; 1, Depot for R10933

COMP = (CENTR_33) ; 2, Central CMT for R10933

COMP = (PERIP_33) ; 3, Peripheral CMT for R10987

COMP = (DEPOT_87) ; 4, Depot for R10987

COMP = (CENTR_87) ; 5, Central CMT for R10987

COMP = (PERIP_87) ; 6, Peripheral CMT for R10987

$PK

MAGEBL = 45 ; Median baseline age (y)

MWGTBL = 81.6 ; Median baseline body weight (kg)

MBMIBL = 28.4 ; Median baselineBMI (kg/m^2)

MALBBL = 43 ; Median baseline albumin (g/L)

MASTBL = 24 ; Median baseline AST (IU/L)

MALTBL = 25 ; Median baseline ALT (IU/L)

MCRCLBL = 124 ; Median baseline CrCL (mL/min)

MBILIBL = 6.8 ; Median baseline total bilirubin

MVRQNP = 6.4 ; Median viral load

WGTBLREF = 70 ; reference body weight (kg)

MCRP = 5.48 ; Median CRP

MNLR = 2.11 ; Median NLR

MINT6 = 10.2 ; Median IL-6

MINT8 = 29.5 ; Median IL-8

;-- Define covariates:

AGEBL = AGEBL1

IF (AGEBL.LE.0) THEN

AGEBL = 0.01

ENDIF

ALBBL = ALBBL1

IF (ALBBL.LT.0) THEN

ALBBL = MALBBL

ENDIF

ALB = ALB1

IF (ALB.EQ.0) THEN

ALB = 0.001

ELSEIF (ALB.LT.0) THEN

ALB = MALBBL

ENDIF

SEXF = 0

IF (SEXN.EQ.2) SEXF = 1

RACW = 0

IF (RACEN.EQ.1) RACW = 1

HEPMILD = HEPMILD1

IF (HEPMILD1.LT.0) HEPMILD = 0

SERPOS = 0

IF (SERBLN.EQ.2) SERPOS = 1

PEDLT6 = 0

IF (AGECTN.LT.4) PEDLT6=1

; --- disease severity

OXYSTAT1 = 0

IF (OXYBL.EQ.1) OXYSTAT1 = 1 ; low flow O2

OXYSTAT2 = 0

IF (OXYBL.EQ.2.OR.OXYBL.EQ.3) OXYSTAT2 = 1 ; high flow O2 / ventilation

VRQNP = VRQNP1

IF (VRQNP.EQ.0) THEN

VRQNP = 0.001

ELSEIF (VRQNP.LT.0) THEN

VRQNP = 6.4

ENDIF

CRP = CRP1

IF (CRP.EQ.0) THEN

CRP = 0.001

ELSEIF (CRP.LT.0) THEN

CRP = MCRP

ENDIF

INT8 = INT81

IF (INT8.EQ.0) THEN

INT8 = 0.001

ELSEIF (INT8.LT.0) THEN

INT8 = MINT8

ENDIF

NLR = NLR1

IF (NLR.EQ.0) THEN

NLR = 0.001

ELSEIF (NLR.LT.0) THEN

NLR = MNLR

ENDIF

CL1COV = ((AGEBL/MAGEBL)**THETA(15))*(1+SEXF*THETA(16))*(1+RACW*THETA(17))*((ALB/MALBBL)**THETA(18))*(1+HEPMILD*THETA(19))*((VRQNP/MVRQNP)**THETA(20))*(1+SERPOS*THETA(21))*((NLR/MNLR)**THETA(24))*(1+OXYSTAT1*THETA(25))*(1+OXYSTAT2*THETA(26))

V2COV = ((AGEBL/MAGEBL)**THETA(27))*(1+SEXF*THETA(28))*(1+RACW*THETA(29))*((ALB/MALBBL)**THETA(30))*(1+HEPMILD*THETA(31))*((VRQNP/MVRQNP)**THETA(32))*(1+SERPOS*THETA(33))*((NLR/MNLR)**THETA(36))

CL2COV = ((AGEBL/MAGEBL)**THETA(15))*(1+SEXF*THETA(38))*(1+RACW*THETA(17))*((ALB/MALBBL)**THETA(40))*(1+HEPMILD*THETA(19))*((VRQNP/MVRQNP)**THETA(20))*(1+SERPOS*THETA(21))*((NLR/MNLR)**THETA(24))*(1+OXYSTAT1*THETA(25))*(1+OXYSTAT2*THETA(26))

V5COV = ((AGEBL/MAGEBL)**THETA(49))*(1+SEXF*THETA(50))*(1+RACW*THETA(51))*((ALB/MALBBL)**THETA(30))*(1+HEPMILD*THETA(53))*((VRQNP/MVRQNP)**THETA(54))*(1+SERPOS*THETA(55))*((NLR/MNLR)**THETA(58))

TVCL1COV = THETA(1)*(WGTBL/MWGTBL)**(THETA(13)*(1-PEDLT6) + 0.75*PEDLT6)*CL1COV

TVCL1 = TVCL1COV

IF (STDY.EQ.2067) THEN

TVCL1 = TVCL1COV*(CRP/MCRP)**THETA(22)

ENDIF

IF (STDY.EQ.2066) THEN

TVCL1 = TVCL1COV*(CRP/MCRP)**THETA(22)*(INT8/MINT8)**THETA(23)

ENDIF

CL1 = TVCL1*EXP(ETA(1))

TVV2COV = THETA(2)*(WGTBL/MWGTBL)**(THETA(14)*(1-PEDLT6) + 1*PEDLT6)*V2COV

TVV2 = TVV2COV

IF (STDY.EQ.2067) THEN

TVV2 = TVV2COV*(CRP/MCRP)**THETA(34)

ENDIF

IF (STDY.EQ.2066) THEN

TVV2 = TVV2COV*(CRP/MCRP)**THETA(34)*(INT8/MINT8)**THETA(35)

ENDIF

V2 = TVV2*EXP(ETA(2))

TVQ1 = THETA(3)

Q1 = TVQ1

TVV3 = THETA(4)

V3 = TVV3*EXP(ETA(2))

TVCL2COV = THETA(5)*(WGTBL/MWGTBL)**(THETA(13)*(1-PEDLT6) + 0.75*PEDLT6)*CL2COV

TVCL2 = TVCL2COV

IF (STDY.EQ.2067) THEN

TVCL2 = TVCL2COV*(CRP/MCRP)**THETA(22)

ENDIF

IF (STDY.EQ.2066) THEN

TVCL2 = TVCL2COV*(CRP/MCRP)**THETA(22)*(INT8/MINT8)**THETA(45)

ENDIF

CL2 = TVCL2*EXP(ETA(1))

TVV5COV = THETA(6)*(WGTBL/MWGTBL)**(THETA(14)*(1-PEDLT6) + 1*PEDLT6)*V5COV

TVV5 = TVV5COV

IF (STDY.EQ.2067) THEN

TVV5 = TVV5COV*(CRP/MCRP)**THETA(56)

ENDIF

IF (STDY.EQ.2066) THEN

TVV5 = TVV5COV*(CRP/MCRP)**THETA(56)*(INT8/MINT8)**THETA(57)

ENDIF

V5 = TVV5*EXP(ETA(2))

TVQ2 = THETA(7)

Q2 = TVQ2

TVV6 = THETA(8)

V6 = TVV6*EXP(ETA(2))

TVKA1 = THETA(9)

KA1 = TVKA1*EXP(ETA(3))

TVKA2 = THETA(10)

KA2 = TVKA2*EXP(ETA(3))

TVF1 = THETA(11)

IF (AGECTN.LT.7) THEN

;TVF1 = EXP(THETA(59))/(1+EXP(THETA(59)))

TVF1 = THETA(59)

ENDIF

F1 = TVF1

TVF4 = THETA(12)

IF (AGECTN.LT.7) THEN

;TVF4 = EXP(THETA(60))/(1+EXP(THETA(60)))

TVF4 = THETA(60)

ENDIF

F4 = TVF4;DERIVED PARAMETERS

K20 = CL1/V2

K23 = Q1/V2

K32 = Q1/V3

K50 = CL2/V5

K56 = Q2/V5

K65 = Q2/V6

S2 = V2/1000 ; AMT = g, conc = mg/L

S5 = V5/1000 ; AMT = g, conc = mg/L

IF (ROUTN.EQ.2) THEN

ETASXI(3) = 1 ; Exclude IV arms in ETA shrinkage assessment for KA

ENDIF

$DES

DADT(1) = -KA1*A(1)

DADT(2) = KA1*A(1) - K20*A(2) - K23*A(2) + K32*A(3)

DADT(3) = K23*A(2) - K32*A(3)

DADT(4) = -KA2*A(4)

DADT(5) = KA2*A(4)-K50*A(5) - K56*A(5) + K65*A(6)

DADT(6) = K56*A(5) - K65*A(6)

$ERROR (OBSERVATION ONLY)

CP_933 = A(2)/S2

CP_987 = A(5)/S5

IF(ORDER.EQ.1) THEN

IPRED = LOG(CP_933)

W = 1

IRES = DV - IPRED

IWRES = IRES/W

Y = IPRED + W * EPS(1)

ENDIF

IF (ORDER.EQ.2) THEN

IPRED = LOG(CP_987)

W = 1

IRES = DV - IPRED

IWRES = IRES/W

Y = IPRED + W * EPS(2)

ENDIF

$ESTIMATION METHOD=1 INTER NOABORT MAXEVAL=9999 NSIG=2 SIGL=6 PRINT=5 SORT

$COV PRINT=E UNCONDITIONAL

$TABLE ID ROWID STDY TRTAN CMT NTIM TIME TAD DV IPRED PRED IWRES CWRES BLQ MDV EVID TVCL1 CL1 TVV2 V2 Q1 V3 KA1 F1 TVCL2 CL2 TVV5 V5 Q2 V6 F4 KA2 WGTBL AGEBL AGECTN ALB ALBBL SEXN RACEN VRQNP SERBLN OXYBL LIVRFNCN CRP NLR INT8 CRCLBL CL1COV V2COV CL2COV V5COV ETA1 ETA2 ETA3 NOPRINT NOAPPEND ONEHEADER FILE=run3021b.tbl FORMAT=s1PE13.7

$TABLE ID STDY TRTAN CMT NTIM TIME DV IPRED PRED IWRES CWRES BLQ MDV EVID ETA1 ETA2 ETA3 NOPRINT NOAPPEND ONEHEADER FILE=sdtab_run3021b FORMAT=s1PE13.7

$TABLE ID STDY TRTAN CMT TVCL1 CL1 TVV2 V2 Q1 V3 KA1 F1 TVCL2 CL2 TVV5 V5 Q2 V6 F4 KA2 ETA1 ETA2 ETA3 NOPRINT NOAPPEND ONEHEADER FILE=patab_run3021b FORMAT=s1PE13.7

$TABLE ID STDY TRTAN CMT ETA1 ETA2 AGEBL WGTBL BMIBL ALBBL ASTBL ALTBL CRCLBL VRQNP NOPRINT NOAPPEND ONEHEADER FILE=cotab_run3021b FORMAT=s1PE13.7

$TABLE ID STDY TRTAN CMT ETA1 ETA2 SEXN SERBLN RACEN LIVRFNCN NOPRINT NOAPPEND ONEHEADER FILE=catab_run3021b FORMAT=s1PE13.7

Supplemental Table S1. Summary of studies included in the population PK analysis

| **Study registration and protocol number** | **Phase** | **Title/design/population** | **Treatment groups** | **N** | **PK sampling schedule** |
| --- | --- | --- | --- | --- | --- |
| NCT04426695 | 1/2/3 | A master protocol assessing the safety, tolerability, and efficacy of anti-spike (S) SARS-CoV-2 monoclonal antibodies for the treatment of hospitalized patients with COVID-19 | CAS+IMD IV 2.4 g or 8.0 g single dose or placebo | 1303 | Phase 1: pre-dose, post-dose^a^, days 3, 5, 7, 15 and 29. When applicable, at discharge before day 29.  Phase 2/3: pre-dose, post-dose, day 15, day 29. When applicable, at discharge before day 29. |
| NCT04425629 | 1/2/3 | A master protocol assessing the safety, tolerability, and efficacy of anti- spike(s) SARS-CoV-2 monoclonal antibodies for the treatment of ambulatory adult and pediatric patients with COVID-19 | Adults: CAS+IMD 1.2 g, 2.4 g or 8 g IV or placebo  Treatment groups for pediatrics are presented in Supplemental Table S2. | 4309 | Phase 1: pre-dose, post-dose^a^,  days 3, 5, 7, 15 and 29. When applicable, at discharge before day 29.  Phase 2: pre-dose, post-dose^a^ and day 29.  Phase 3 (patients ≥18 years): pre-dose, post-dose^a^, days 29 and 120  Phase 3 (patients <18 years): pre-dose, post-dose^a^, days 3, 7, 15, 22, 29, 90 and 120 |
| NCT04452318 | 3 | A randomized, double-blind, placebo-controlled study assessing the efficacy and safety of anti-spike SARS-CoV-2 monoclonal antibodies in preventing SARS-CoV-2 infection in household contacts of individuals infected with SARS-CoV-2 | CAS+IMD 1.2 g SC or placebo  Treatment groups for pediatrics are presented in Supplemental Table S2. | 287 | Adult/adolescents:  Sentinel group: pre-dose, Days 2, 4, 8, 15, 22, 29, 57, 85, 113, 141, 169, 197, 225 and early termination visit when applicable.  Safety group: pre-dose, days 29, 57, 113, 169, 225 and early termination visit when applicable. |
| NCT04519437 | 1 | A Phase 1, randomized, double-blind, placebo-controlled study assessing the safety, tolerability, pharmacokinetics, and immunogenicity of repeated subcutaneous doses of anti-spike SARS-CoV-2 monoclonal antibodies (REGN10933+REGN10987) in adult volunteers | CAS+IMD 1.2 g SC or placebo Q4W up to 6 doses | 724 | Pre-dose, days 8, 29, 57, 85, 113, 141, 148, 169, 225, 281, and 365 |
| NCT04666441 | 2 | A Phase 2 study assessing the virologic efficacy of REGN10933+REGN10987 across different dose regimens in adult outpatients with SARS-CoV-2 infection | CAS+IMD IV 2.4, 1.2, 0.6 or 0.3 g single dose or placebo  CAS+IMD SC 1.2 or 0.6 g single dose or placebo | 967 | Pre-dose, post-dose^a^, days 3, 5, 7 and 120 |
| NCT05092581 | 1b | A phase 1b, open-label, single dose study assessing the pharmacokinetics, safety, tolerability, and efficacy of intravenous anti-spike(s) SARS-CoV-2 monoclonal antibodies (CAS+IMD) for the treatment of pediatric patients hospitalized due to COVID-19 | Single IV body weight-based dose equivalent to CAS+IMD 2.4 g or 8.0 g IV adult dose (Supplemental Table S2) | 1 | Pre-dose, post-dose^a^, days 7, 29, 57 and 113 |
| NCT04992273 | 2a | A phase 2a, open-label study assessing pharmacokinetics, safety, tolerability, and immunogenicity of single-dose subcutaneous anti-spike(s) SARS-CoV-2 monoclonal antibodies (CAS+IMD) in high-risk pediatric subjects under 12 years of age | Single SC body weight-based dose equivalent to CAS+IMD 1.2 g SC adult dose (Supplemental Table S2) | 7 | Pre-dose, days 2, 4, 8, 15, 29, 57, 85 and 113 |

^a^For IV, within 60 min after the end of infusion; for SC, at least 60 min after study drug administration*.* CAS+IMD, casirivimab plus imdevimab; COVID-19, coronavirus disease 2019; IV, intravenous; N, number of participants; PK, pharmacokinetic; Q4W, every 4 weeks; REGN10933, casirivimab; REGN10987, imdevimab; SARS-CoV-2, severe acute respiratory syndrome coronavirus 2; SC, subcutaneous.

Supplemental Table S2. Treatment groups for pediatric participants based on body weight equivalent to CAS+IMD 1.2 or 2.4 g IV or 1.2 g SC

| Body weight group |  |  |  |  |
| --- | --- | --- | --- | --- |
| ≥40 kg | 1.2 g IV | 2.4 g IV | 1.2 g SC | 8g IV |
| ≥20 kg to <40 kg | 450 mg IV | 900 mg IV | 792 mg SC | 3 g IV |
| ≥10 kg to <20 kg | 224 mg IV | 450 mg IV | 408 mg SC | 1600 mg |
| ≥5 kg to <10 kg | 120 mg IV | 240 mg IV | 144 mg SC | 700 mg |

CAS+IMD, casirivimab plus imdevimab; IV, intravenous; SC, subcutaneous.

Note: The doses in the table are the total amount of CAS+IMD, which comprised of equal amount of each monoclonal antibody.

Supplemental Fig. S1 Diagnostic plots for the final population pharmacokinetics model^a^: (a) Log-transformed casirivimab and (b) Log-transformed imdevimab

**(a)**
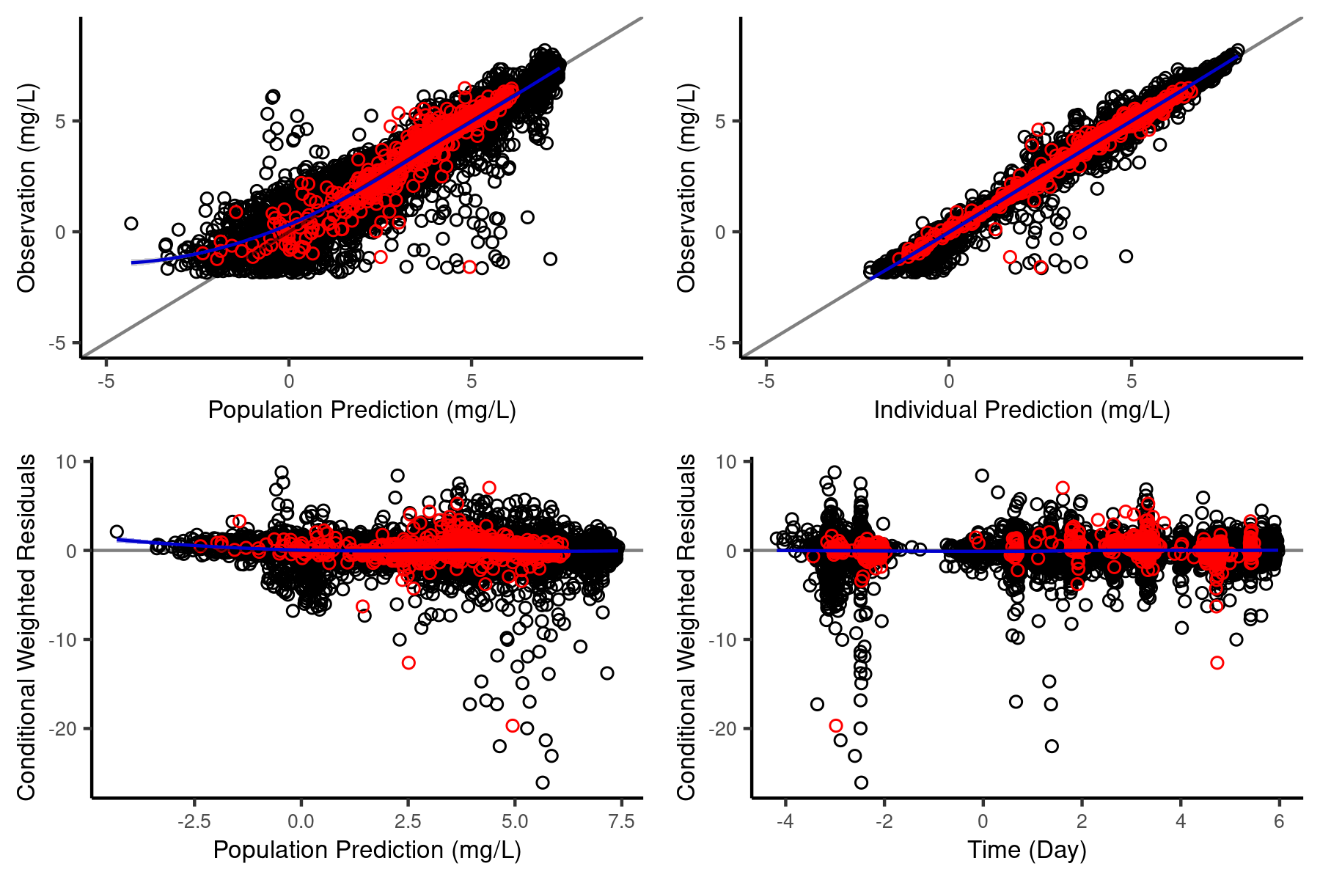

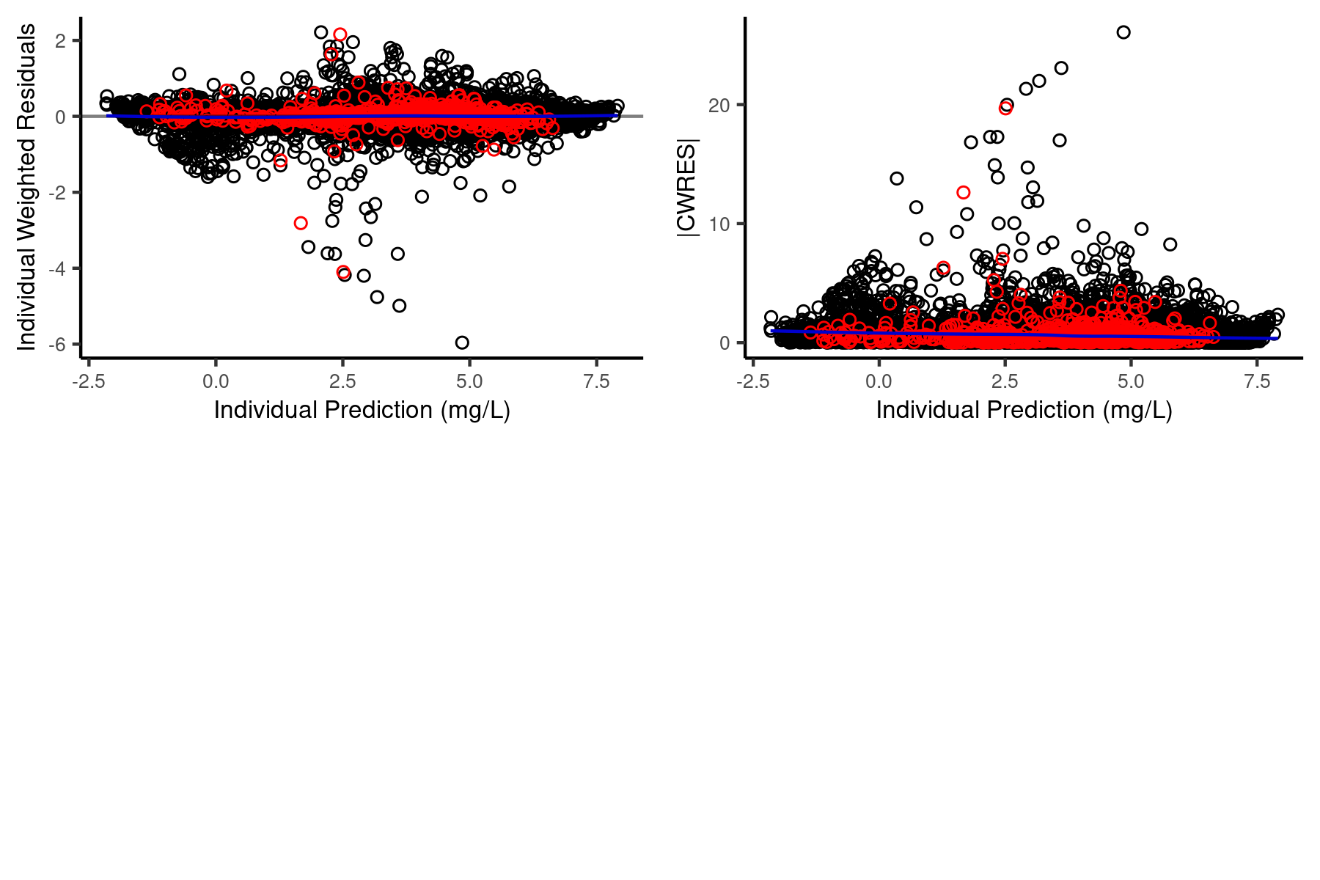


**(b)**
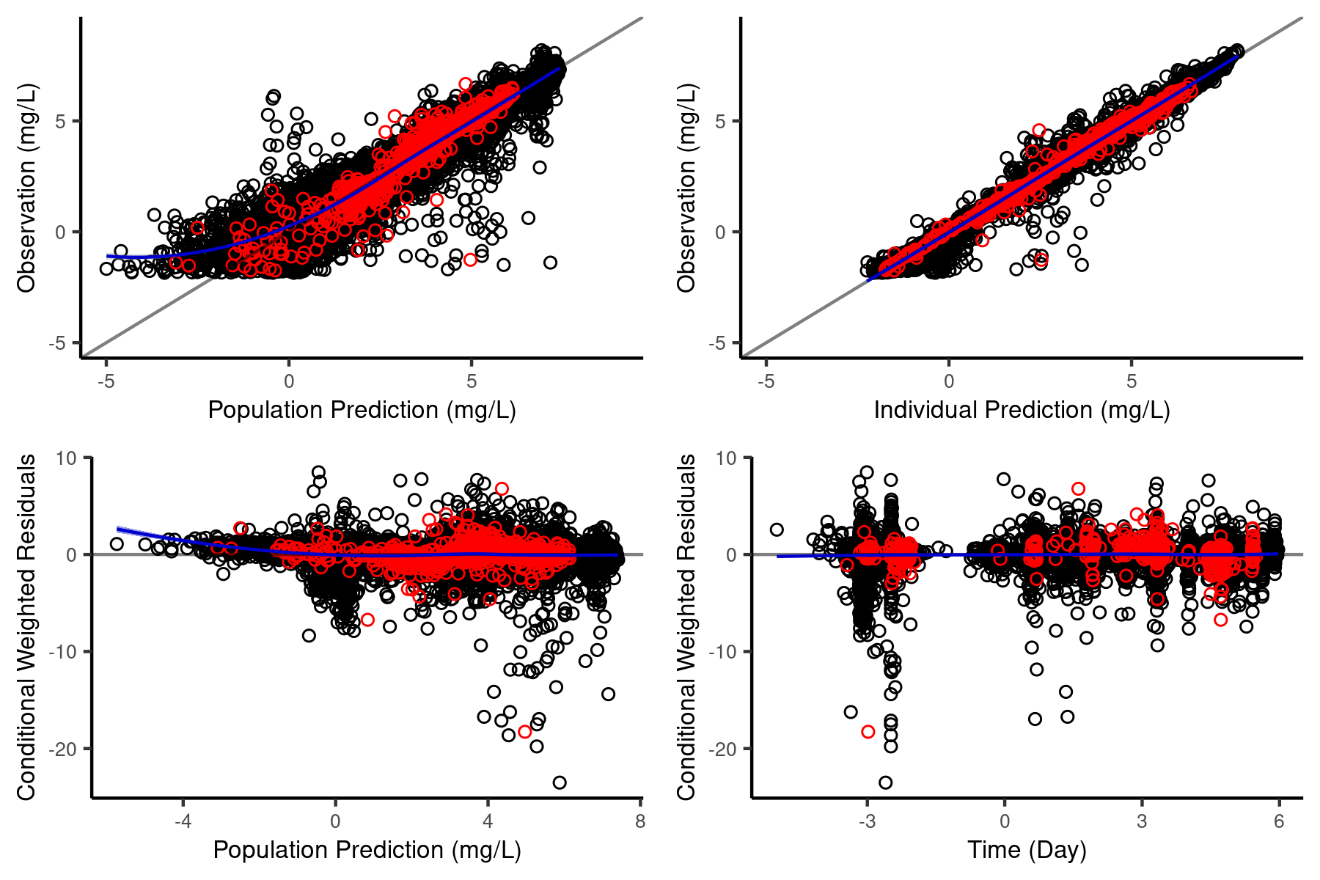

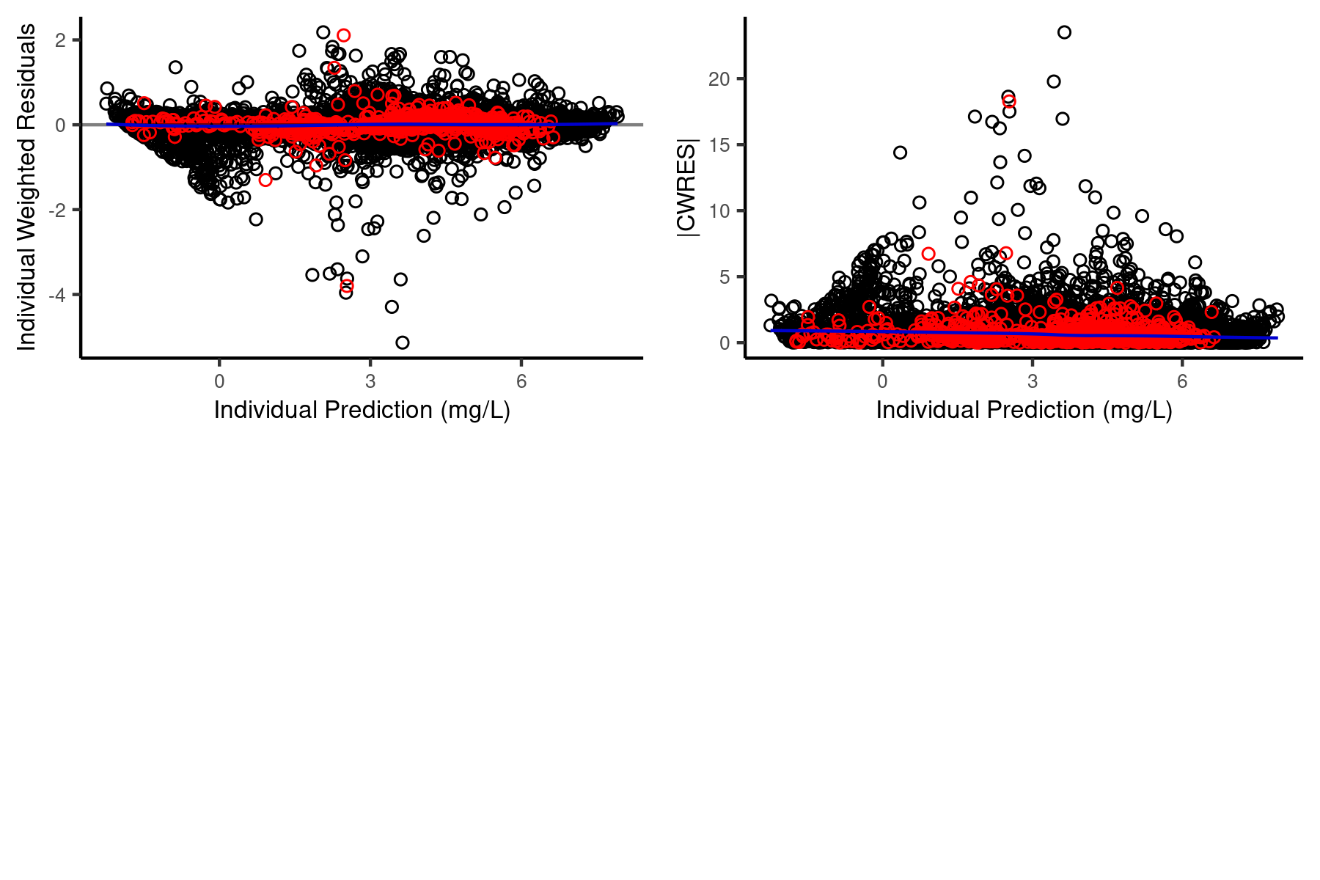


^a^Red symbols represent data from pediatrics <18 years of age. Symbols represent individual observations. The blue lines are smoothness (loess). The solid diagonal and horizontal lines are the line of identity and a zero reference line. Observations, population predictions, individual predictions and time are log-transformed.

Supplemental Fig. S2. Diagnostic plots for the final population pharmacokinetics model^a^: (a) casirivimab and (b) imdevimab


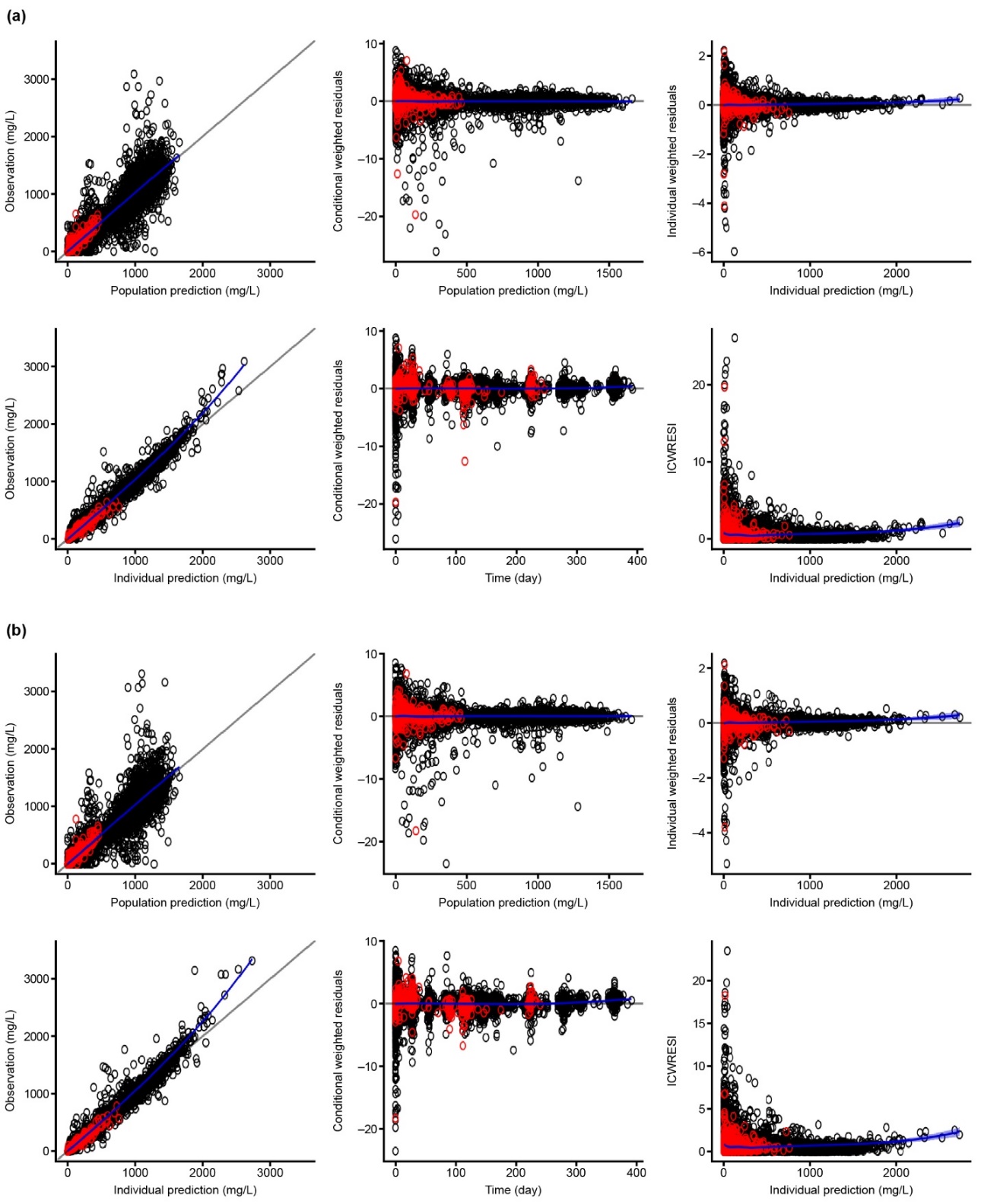


^a^Red symbols represent data from pediatrics <18 years of age. Symbols represent individual observations. The blue lines are smoothness (loess). The solid diagonal and horizontal lines are the line of identity and a zero reference line.

Supplemental Fig. S3. Prediction-corrected visual predictive check plots for the final population pharmacokinetics model, in log_10_ scale: (a) SC route and (b) IV route

**(a)**
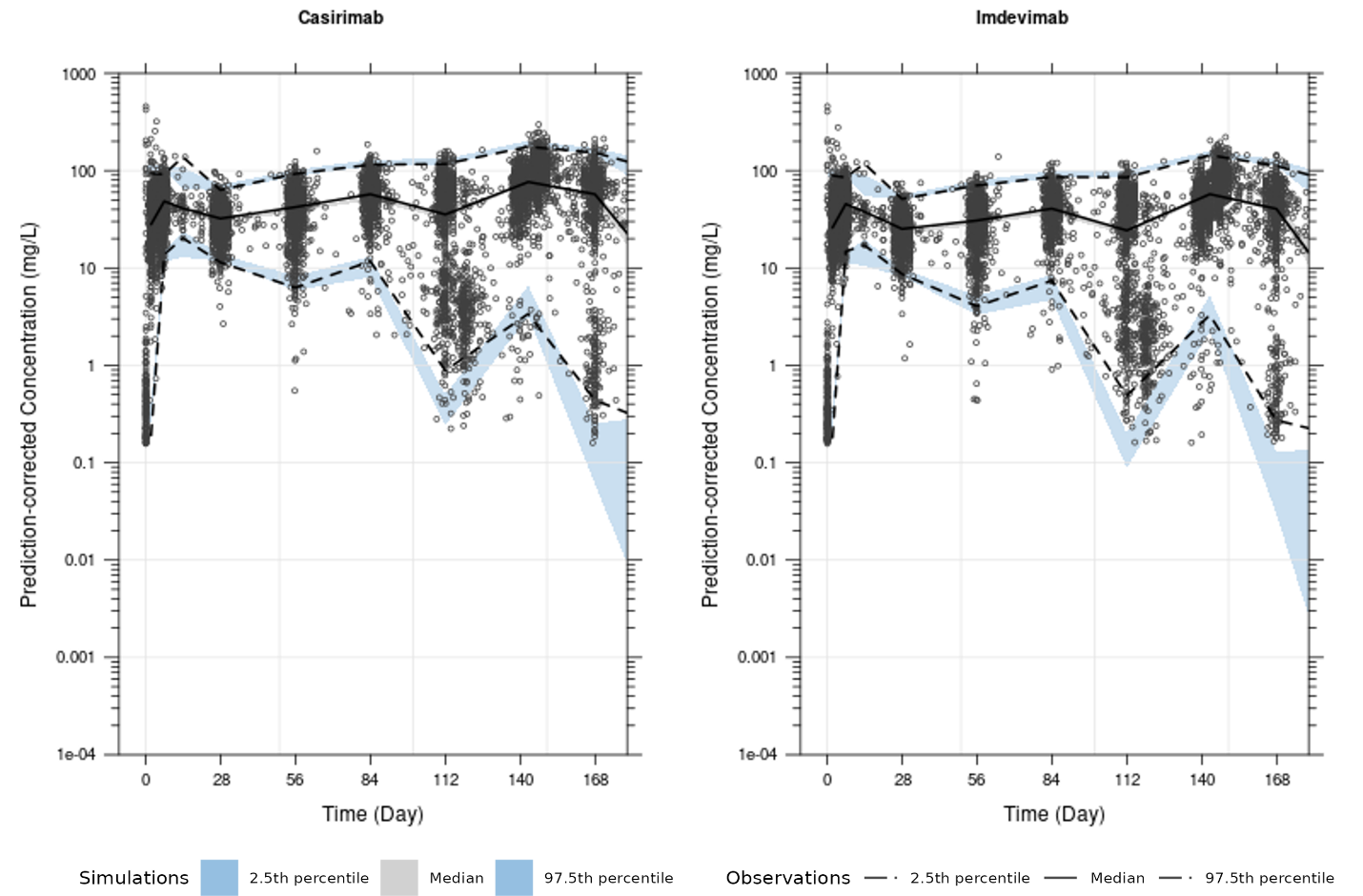


**(b)

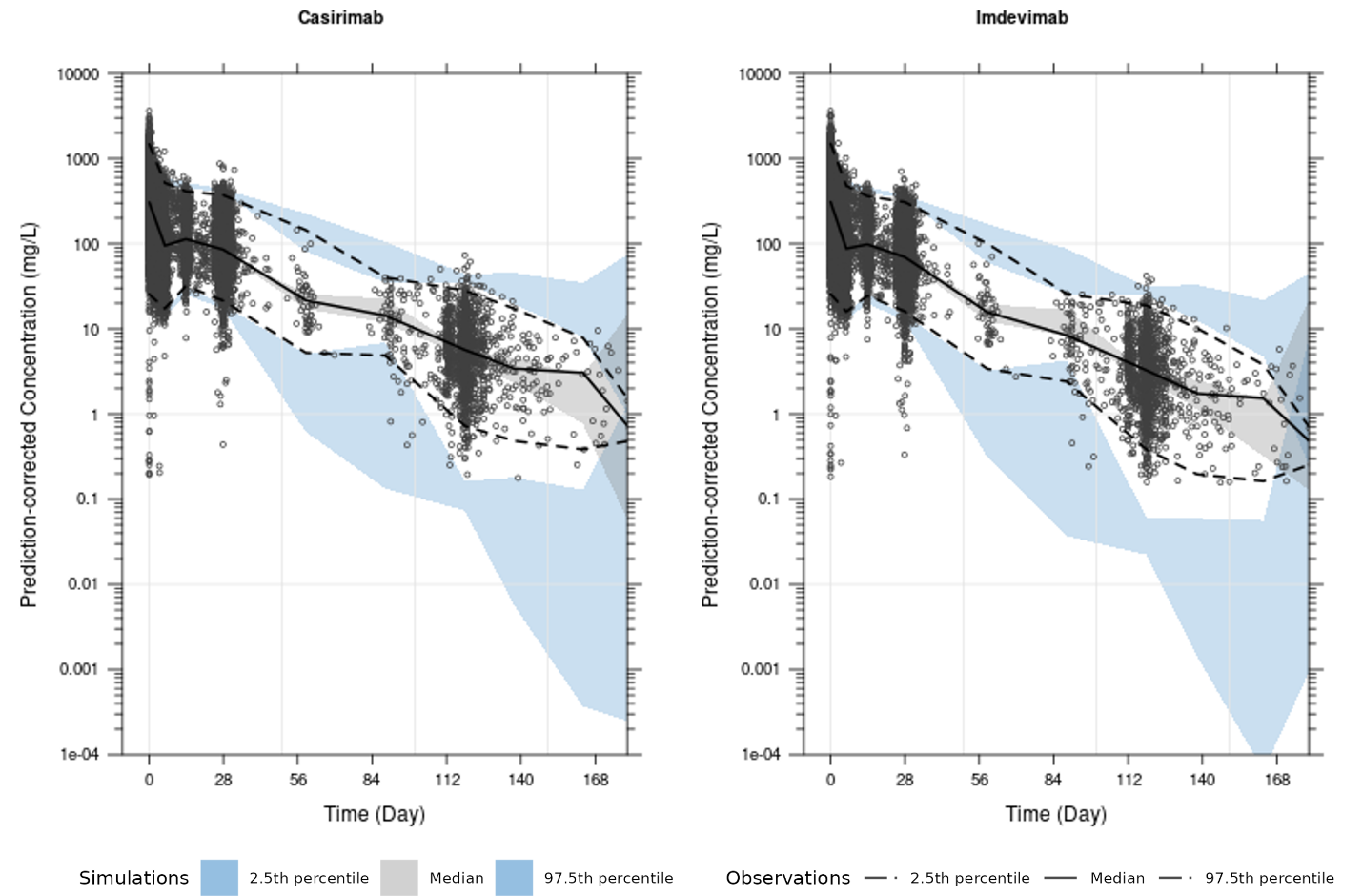
**

Supplemental Fig. S4. Prediction-corrected visual predictive check plots based on time after dose for the final population pharmacokinetics model following SC administration route, in log_10_ scale

**
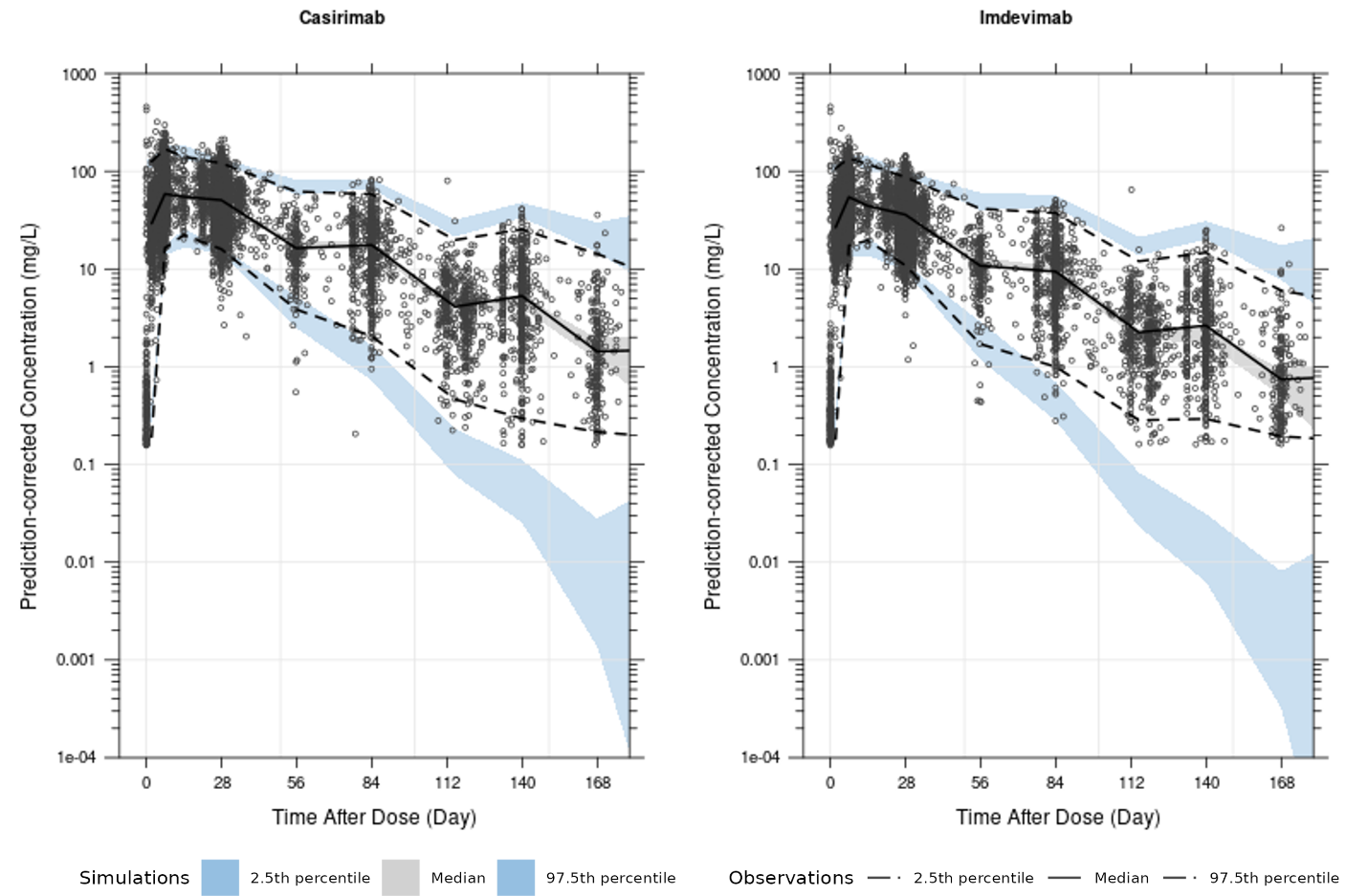
**

SC, subcutaneous.
